# Global Gridded Crop Production Dataset at 10 km Resolution from 2010 to 2020

**DOI:** 10.1038/s41597-024-04248-2

**Published:** 2024-12-18

**Authors:** Xingli Qin, Bingfang Wu, Hongwei Zeng, Miao Zhang, Fuyou Tian

**Affiliations:** 1 Key Laboratory of Remote Sensing and Digital Earth, Aerospace Information Research Institute, Chinese Academy of Sciences, Beijing, 100101 China; 2 College of Resources and Environment, University of Chinese Academy of Sciences, Beijing, 100049 China

**Keywords:** Agriculture, Environmental impact

## Abstract

The global gridded crop production dataset at 10 km resolution from 2010 to 2020 (GGCP10) for maize, wheat, rice, and soybean was developed to address limitations of existing datasets characterized by coarse resolution and discontinuous time spans. GGCP10 was generated using a series of adaptively trained data-driven crop production spatial estimation models integrating multiple data sources, including statistical data, gridded production data, agroclimatic indicator data, agronomic indicator data, global land surface satellite products, and ground data. These models were trained based on agroecological zones to accurately estimate crop production in different agricultural regions. The estimates were then calibrated with regional statistics for consistency. Cross-validation results demonstrated the models’ performance. GGCP10’s accuracy and reliability were evaluated using gridded, survey, and statistical data. This dataset reveals spatiotemporal distribution patterns of global crop production and contributes to understanding mechanisms driving changes in crop production. GGCP10 provides crucial data support for research on global food security and sustainable agricultural development.

## Background & Summary

Information on crop production plays a critical role in global food security and sustainable agricultural development^1,2^. Four major crops, namely maize, wheat, rice, and soybean, contribute to over 64% of the world’s caloric intake^2^. The increased demand for food, coupled with global climate change and population growth, puts immense pressure on countries to secure food supplies^3,4^. Thus, there is a growing need to gain insights into the distribution of food production for sustainable agriculture^5–7^. Therefore, it is critical to develop a time-series, high-precision dataset of the global crop production distribution for research on food production and consumption, policymaking, optimization of resource use, and planning for sustainable agricultural development^8^.

Currently available global crop production datasets, while valuable, have limitations in temporal and spatial resolution (Table 1). The Spatial Production Allocation Model (SPAM)^9^ covers only three years (2000, 2005, and 2010) at a 5 arc-minute resolution. M3-Crops^10^, while offering data for 175 crops, is limited to the year 2000. The global dataset of historical yields for major crops (GDHY)^11^, covering 1981–2016, provides annual data but at a coarse 30 arc-minute resolution. The Global Gridded Crop Model Intercomparison (GGCMI) phase 1 dataset^12^ spans 1901–2012 but only at 10-year intervals. The Global Agro-Ecological Zones (GAEZ) dataset^13,14^ offers data for 2000, 2010, and 2015 at 5 arc-minutes resolution. Additionally, these datasets primarily rely on statistical disaggregation or model simulations, which may not fully account for spatial heterogeneity and intra-annual environmental variations. Consequently, the different research purposes and technical limitations of these datasets result in insufficient temporal and spatial resolution and coverage. Furthermore, the lack of temporal continuity and timeliness of the data have failed to capture the effects of drastic global climate changes that have occurred in the past decade^15,16^. Therefore, there is a global shortage of temporally continuous, high-resolution, and gridded crop production datasets.

**Table 1:** Overview of currently available global crop production datasets.

Dataset name	Time coverage	Spatial resolution	Crop type coverage	Method description
SPAM^9^	2000 2005 2010	5 arc-minutes	42 crops	Disaggregates national/subnational crop statistics to grid cells using a cross-entropy approach, incorporating data on cropland, irrigation, crop suitability, population, and market access.
M3-Crops^10^	2000	5 arc-minutes	175 crops	Distributes national and subnational crop statistics to grid cells proportionally based on satellite-derived cropland data and crop-specific suitability.
GDHY^11^	1981–2016 (annual)	30 arc-minutes	4 crops	Combines census statistics with satellite-derived NPP to estimate grid-cell yields, using crop calendars and multi-cropping information to differentiate cropping seasons.
GGCMI phase 1^12^	1901–2012 (10-year intervals)	30 arc-minutes	15 crops (4 crops in priority 1 and 11 crops in priority 2)	Global gridded crop model simulations using harmonized inputs, producing crop yields and other variables for multiple crops, management scenarios, and climate datasets.
GAEZ^13,14^	2000 2010 2015	5 arc-minutes	26 crops	Apply optimization techniques to downscale national crop statistics to grid cells based on land suitability, irrigation potential, and agro-climatic conditions, considering multiple cropping and management levels.

The era of remote-sensing big data has produced a wealth of global observational data, which offer new opportunities to address the spatial distribution of crop production. These massive and diverse remote sensing datasets contain rich information related to crop production, such as climate, land cover, and vegetation growth conditions^17,18^. Additionally, ground information, such as soil characteristics and topographic conditions, serves as an essential reference for estimating crop production. Machine learning techniques have exhibited good performance in predicting crop yields and production^19–21^, thereby revealing deep correlations between crop production and various observation indicators in recent years. Hence, integrating information from multiple sources and using machine learning models to uncover the intrinsic relationships between crop production and observation indicators to obtain accurate spatial distributions of crop production is a feasible approach^22,23^.

**Fig. 1:**
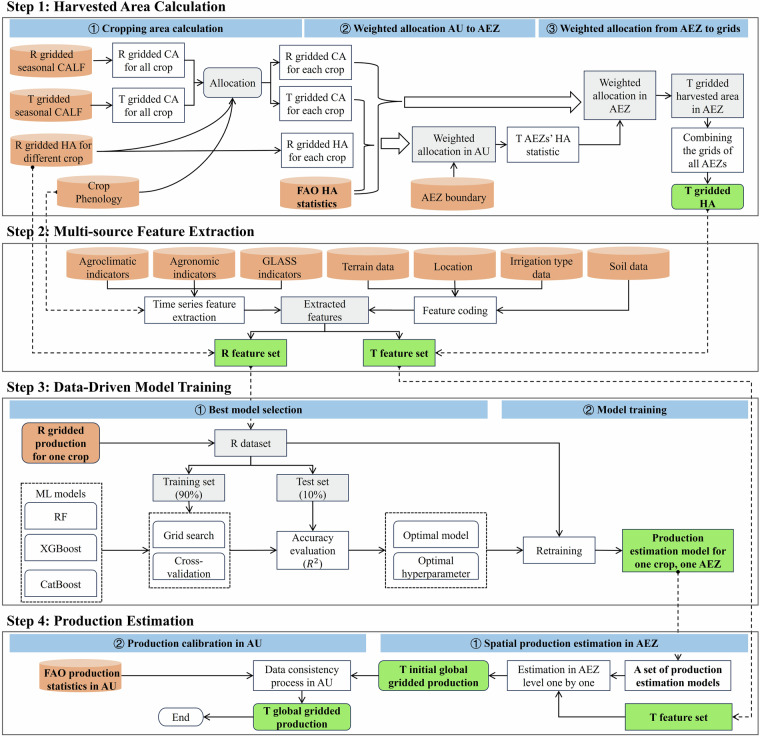
Methodological framework for generating the GGCP10 global gridded crop production dataset. (R: Reference year; T: Target year; CA: Cropping Area; HA: Harvested Area; AU: Administrative Unit; AEZ: Agro-Ecological Zone).

A global gridded dataset of maize, wheat, rice, and soybean production was constructed at a 10 km resolution from 2010 to 2020^24^. The development of the GGCP10 dataset involved a comprehensive process integrating multiple data sources and employing advanced machine learning techniques to estimate crop production at the grid-cell level. The overall workflow consisted of four main steps: harvested area calculation, multi-source feature extraction, data-driven model training, and production estimation, as shown in Fig. 1. This data-driven production estimate model incorporated multiple source datasets, including statistical data, gridded production data, agroclimatic indicator data, agronomic indicator data, global land surface satellite products, and ground data. Importantly, this approach utilized time-series data of environmental factors and crop growth indicators, allowing to capture intra-annual variations in climate and crop conditions that significantly influence crop production. A set of data-driven models was developed based on agroecological zones and multiple factors, capable of capturing the inherent correlations between crop production, harvested area, and other indicators to achieve high prediction accuracy. The dataset underwent rigorous examination through preprocessing and consistency checks to ensure data accuracy and reliability.

With its unique spatiotemporal continuity and high-resolution characteristics, the GGCP10 dataset offers broad application prospects in fields such as agricultural production monitoring, food security analysis, and agricultural policy formulation at regional to global scales. These applications were previously constrained by aggregated, limited, and inconsistent agricultural statistical data^9,25^. Moreover, integrating GGCP10 with other global gridded datasets (e.g., population and GDP) enables a more comprehensive analysis of the interactions between agricultural production and socioeconomic development, thereby providing crucial support for achieving sustainable development goals.

## Methods

Here we present a comprehensive description of the multi-source data and data-driven methods used to generate the GGCP10 dataset.

### Input data

To construct the spatial production estimation model, we collected data from multiple sources, including Food and Agriculture Organization of the United Nations (FAO) statistical data, GAEZ+ 2015 annual crop data, CropWatch crop phenology data, CropWatch global eco-agricultural zoning, SoilGrids soil texture data, CropWatch irrigated land distribution data, latitude and longitude, topographic data, CropWatch agroclimatic indicators, CropWatch agronomic indicators, and Global Land Surface Satellite (GLASS) remote sensing data products.

1. **FAO Statistical Data**. This study employed crop-harvested areas and production data for various countries from FAOSTAT (https://www.fao.org/faostat/en/#data). The data included the harvested area and production information for four major crops (maize, wheat, rice, and soybean), with country as the statistical unit. The data were measured in tons and hectares from 1961 to 2022, with this study utilizing the data from 2010 to 2020.
  The primary role of these data in the study is consistency processing, ensuring that the sum of the estimated harvested areas within each country matches the FAO statistical data, and that the sum of the allocated crop production equals the FAO statistical data. The FAO data offer distinct advantages for constructing a global long time-series crop production dataset: the data sources are authoritative, the time series is lengthy, and official statistics from various countries have been systematically aggregated and verified. Therefore, this study uses FAO national statistical data to calibrate global-scale production estimation results to achieve better cross-regional comparability based on the existing data foundation.
2. **GAEZ+ 2015 Annual Crop Data**. The 2015 GAEZ gridded crop production dataset and gridded crop harvested area dataset^14^ were used as training data for the production estimation model. This choice was based on several factors: its comprehensive global coverage, high spatial resolution, and demonstrated accuracy, all of which align well with our research objectives. Additionally, as our target period is 2010–2020, the 2015 dataset serves as a reliable midpoint reference, allowing us to model both backward and forward from this central year.
  These data are presented in a gridded format with a grid size of 10 km × 10 km. The pixel values in the production and harvested area datasets represent the crop production and harvested areas within each grid. The data were used for four types of crops: maize, wheat, rice, and soybean. The GAEZ+ 2015 dataset has been validated against FAOSTAT reported harvested area, showing high consistency for major crops. For instance, the world’s four staple crops (maize, wheat, rice, and soybean) all have less than 0.5% difference in crop harvested area between FAOSTAT and GAEZ+ 2015. Additionally, when compared with other global gridded datasets, GAEZ+ 2015 demonstrates strong agreement in spatial distribution patterns, particularly for cropland physical area (Coefficient of determination R² $\ge$ 0.9 when aggregated spatially).
3. **CropWatch Crop Phenology Data**. To capture key information on crop growth in different regions more accurately, this study adopted a phenology-based time-window extraction strategy. By delineating the critical time windows of crop growth for different regions and performing time-series feature extraction, the interference of nongrowing season information can be effectively reduced, thereby improving the accuracy of production estimation. Crop phenological data were obtained from the CropWatch System^26^. This dataset, based on extensive field observations and scientific experiments, provides information on the growing and harvesting periods of major crops in major countries at a temporal resolution of 10 days^27^. It is worth noting that although the phenological data used in this study were not updated annually, our goal was to ensure that the extracted features covered the main crop growth time windows rather than obtaining precise phenological timing. Therefore, the temporal resolution and annual representativeness of the existing data are sufficient to meet the research requirements. Using CropWatch phenology data to guide time-series feature extraction, this study can more specifically characterize the crop growth process, which provides important support for improving the explanatory power and generalization ability of the production estimation model.
4. **CropWatch Agro-Ecological Zones**. In this study, we used agroecological zone (AEZs) data from the CropWatch system^26^, which covers 228 agro-ecological zones in 45 countries worldwide, as shown in Fig. 2. These data are based on multiple factors, such as climate, soil, and topography, in different parts of the world, and they comprehensively divide the different agricultural ecological zones. These ecological zones represent regions with similar agricultural production conditions, crop planting patterns, and management patterns and are of great value in understanding and predicting the global distribution of crop production.

**Fig. 2:**
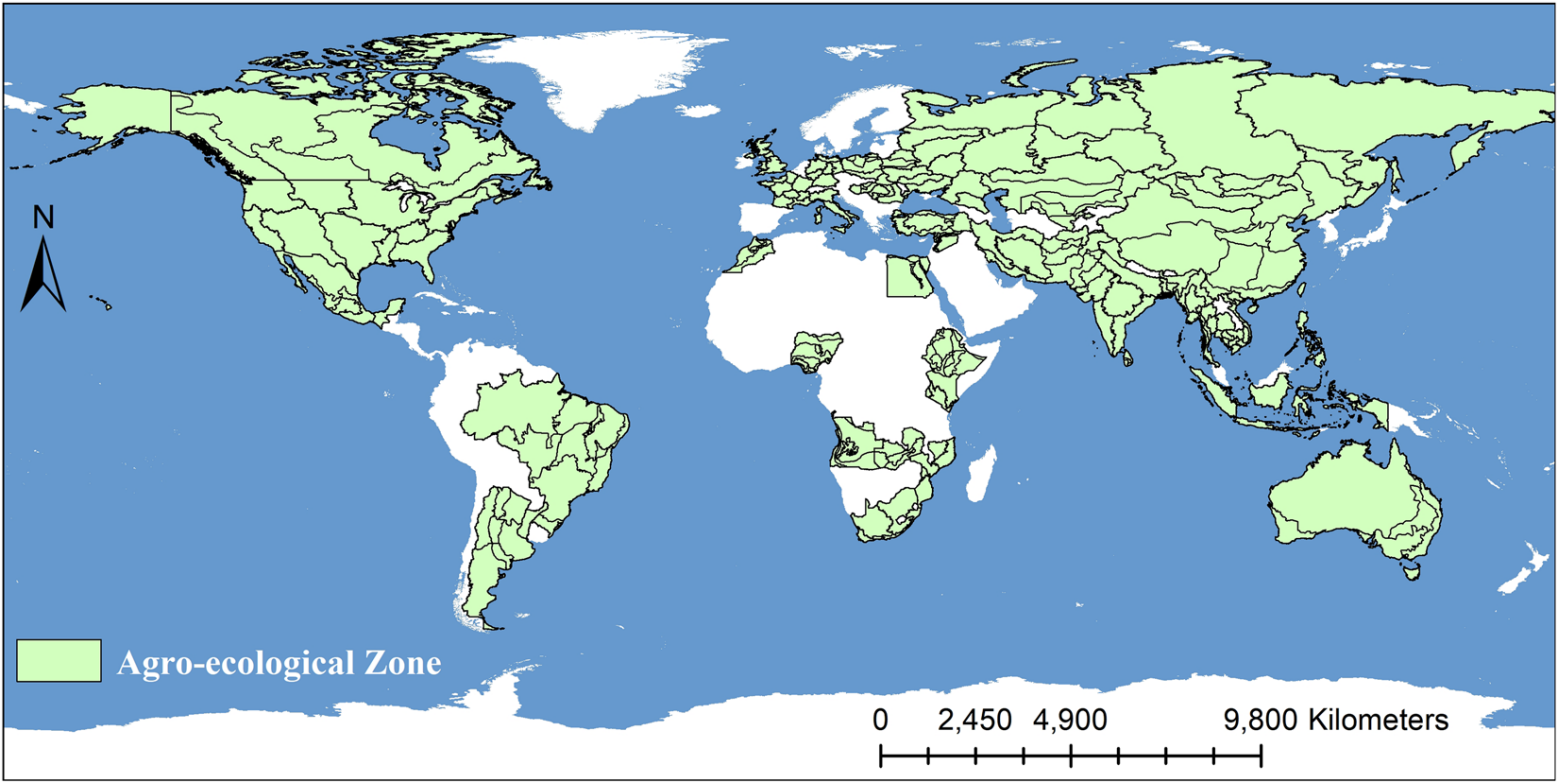
Distribution of 228 agro-ecological zones. Each zone represents the smallest unit for constructing data-driven crop production estimation models.
  One crucial reason for modeling at the agro-ecological zone scale is our aspiration to enable the model to characterize the spatial variation of production within each ecological zone by leveraging the differences in multiple variables within the zone. Diverse ecological conditions often correspond to distinct planting systems and management patterns, and modeling at the AEZ scale allows us to capture these variations more effectively.
  In this study, we used the divided ecological zones as the smallest modelling scale and built corresponding production spatial allocation models for each ecological zone. For countries or regions without subdivided agroecological zones, modelling was performed using the country or region as a homogeneous area.
5. **SoilGrids Soil Texture Data**. This study utilized soil texture data from SoilGrids 2.0 (https://soilgrids.org), a system for global digital soil mapping that employs state-of-the-art machine learning methods to map the spatial distribution of soil properties worldwide^28^. SoilGrids provides soil data at 250 m resolution for six standard depth intervals (0–5 cm, 1–15 cm, 15–30 cm, 30–60 cm, 60–100 cm, 100–200 cm).
  In this study, we specifically used the proportions of silt, sand, and clay in the 0–5 cm soil layer. Soil texture, defined by these proportions, is a key factor influencing the ability of the soil to retain moisture and nutrients, which directly affects crop growth and yield. For example, sandy soils generally have high aeration and drainage, but poor water and nutrient retention, whereas clayey soils have strong water and nutrient adsorption capacities, but relatively poor aeration and drainage. Different crops vary in their adaptability to soil texture. For instance, wheat and soybean are better suited for growing in well-drained sandy loam and loam soils, whereas rice is more suitable for planting in clayey soils with better water retention.
  We incorporated the soil texture data as one of the input features for the model to characterize the potential impact of different regional soil environments on crop growth. By combining soil texture data with other environmental and management factors, the model can learn and infer the spatial distribution patterns of crop production more comprehensively. This is crucial for improving the accuracy of production estimations and understanding the causes of regional production variations.
6. **Global Maximum Irrigation Extent Data (GMIE)**. Irrigation data reflects differences in irrigation management inputs, which greatly influence crop growth and production. Incorporating irrigation information into the spatial production estimation model can help characterize the impact of water management measures on regional crop yields, thereby improving the explanatory power of the model for production spatial distribution patterns^29^.
  In this study, we used global irrigated cropland data from GMIE^30^, which is derived from the irrigation performance during water stress^31^, as one kind input features. These data provide information on the types of irrigation across the world’s croplands, including irrigated and rainfed cropland. Irrigation plays a key role in ensuring stable and high crop production, particularly in arid and water-scarce regions. In our model, the irrigation type data (irrigated, rainfed and unknown) were uniquely coded and transformed into a three-dimensional feature for modelling.
7. **Location Data**. We used latitude and longitude data to represent the geographical location of each sample. Latitude and longitude data are crucial for capturing the influence of geographical location on crop production, such as solar radiation conditions at different latitudes and climatic zone characteristics at different longitudes. However, in the model construction, the latitude and longitude coordinates differ from the Cartesian coordinates. Cartesian coordinates have smooth and uniform variations, whereas latitude and longitude, as polar coordinates, have uneven numerical changes and are unsuitable for the direct expression or measurement of positional or relational changes. Therefore, in this study, we overcome this problem by converting the latitude and longitude polar coordinates into coordinate features in three-dimensional Cartesian coordinates through geospatial encoding. For point $i$, the following formula was used to convert its latitude and longitude coordinates into geospatial encoding features:
  $${g}_{i}=\left[\begin{array}{c}{g}_{{xi}}\\ {g}_{{yi}}\\ {g}_{{zi}}\end{array}\right]=\left[\begin{array}{c}R\,\sin \,{{\rm{\varnothing }}}_{i}\cos {\theta }_{i}\\ R\,\sin \,{\theta }_{i}\\ {R}\,\cos \,{{\rm{\varnothing }}}_{i}\,\sin {\theta }_{i}\end{array}\right]$$
  where ${g}_{i}$ is the geospatial encoding feature of the sample $i$, ${{\rm{\varnothing }}}_{i}$ is the longitude, ${\theta }_{i}$ is the latitude, and *R* is the radius of the Earth. In modeling, we directly set the value of *R* to 1.
  It should be noted that location features are not used to characterize the production differences between agroecological zones but to represent the spatial association of different pixels within the agroecological zones. In the existing gridded analysis framework, if location information is not introduced, the model treats each pixel as an independent individual and ignores the correlation between adjacent pixels in the production composition. By introducing location features, the model can better learn and utilize the spatial autocorrelation between pixels, thereby improving production estimation accuracy. Recent studies have also shown that tree-based ensemble learning algorithms (such as XGBoost) can effectively capture spatial association information contained in location features^21,32,33^.
8. **Terrain Data**. In this study, we incorporated global terrain data including elevation and terrain variation coefficients as essential environmental features to train a crop production prediction model. Topographical factors significantly influence climatic conditions and water flow, which in turn affect crop growth and production. To obtain these terrain data, we utilized the Shuttle Radar Topography Mission (SRTM) digital elevation model (DEM) provided by the United States Geological Survey (USGS)^34^. The SRTM DEM offers near-global coverage at a spatial resolution of approximately 30 m.
  To calculate the terrain variation coefficient (TVC), we first defined a 10 km square kernel, equivalent to approximately 166 pixels, to compute the neighborhood statistics. We then calculated the mean elevation and standard deviation within each 10 km neighborhood. The TVC was derived by dividing the standard deviation by the mean elevation.
  Considering that open water bodies may introduce noise into TVC data, we applied a masking procedure to remove ocean pixels. We utilized the GlobCover^35^ land-cover dataset to identify open-water areas. By converting the open-water class to a binary mask and applying it to the TVC image, we effectively excluded ocean pixels from the analysis.
  All topographic data were standardized to reduce the influence of dimensions and improve the generalizability of the model.
9. **Agroclimatic Indicator Data**. We used agroclimatic indicator data from the CropWatch system, including cumulative precipitation (RAIN), average air temperature (TEMP), photosynthetically active radiation (PAR), and cumulative potential biomass (BIOMASS). These indicators are time-series data, with RAIN, TEMP, and PAR available 36 times a year and BIOMASS available four times a year. The data covers the entire globe and has been continuously updated since 2007, with this study utilizing the data from 2010 to 2020.
  Among these indicators, BIOMASS is a derived indicator calculated by integration of three models, namely Lieth’s “Miami model”^36^ based on RAIN and TEMP, Chikugo model^37^ based on RAIN and PAR and temperature limited NPP model. BIOMASS model is a well-established empirical model that estimates the net primary productivity (NPP) of vegetation on a global scale. It is assumed that vegetation productivity is limited by different climate variables at different regions across the global. For example, it is commonly limited by temperature in cold regions, by radiation in tropical regions and by precipitation in arid regions. The model used the mean annual temperature, annual PAR and annual precipitation as input variables to calculate the potential biomass.
  In this study, the BIOMASS indicator represented the cumulative potential biomass over a reference period (dekad from i to n), expressed as the combined effect of rainfall and temperature during that period. The unit of BIOMASS is grams of dry matter per square meter over the study period.
  These indicators reflect the energy and moisture conditions of agricultural ecosystems. To effectively utilize these data in our model, we used crop phenology data to slice the agro-meteorological indicators into time windows. Within each time window, we calculated the maximum, minimum, standard deviation, and total sum of the indicators as feature inputs for the model. This approach allowed us to characterize the temporal dynamics of the agroclimatic conditions throughout the crop-growing season.
10. **Agronomic Indicator Data**. In this study we used the agronomic indicator data from CropWatch system, including the cropped arable land fraction (CALF) and the maximum vegetation condition index (VCIx). These data are not publicly available and must be obtained through calculations based on time-series normalized difference vegetation index (NDVI) data. These data provide time-series information with four periods per year, covering the entire globe with continuous updates since 2007. We utilized the data spanning from 2010 to 2020, aligning with our study period.
  CALF is the ratio of cropping area to total cultivated area, calculated based on the NDVI. Based on the NDVI peak value of the pixels, the multi-year mean (NDVI_m_), and the standard deviation (NDVI_std_) of the NDVI peaks, a threshold method was used to distinguish between cropped and uncropped arable land^38^. It is worth noting that CALF is not only used as an input feature for data-driven model construction but also plays an important role in characterizing the dynamic changes in cropping areas in harvested area calculation. CALF data is employed to characterize the dynamic changes of cropping area in each grid cell, which helps improve the temporal accuracy of harvested area calculation.
  VCIx was used to describe the historical level of vegetation conditions during the observation period. Based on the Vegetation Condition Index (VCI) proposed by Kogan^39^, the maximum VCI (VCIx) was adopted to describe the optimal crop condition of the current period compared to the historical maximum crop condition using the following equation:
  $${VCIx}=\frac{{{NDVI}}_{\max {\rm{\_}}c}-{{NDVI}}_{\min {\rm{\_}}h}}{{{NDVI}}_{\max {\rm{\_}}h}-{{NDVI}}_{\min {\rm{\_}}h}}$$
  where ${{NDVI}}_{\max {\_c}}$ is the maximum NDVI of a fixed period, and ${{NDVI}}_{\max {\_h}}$ and ${{NDVI}}_{\min {\_h}}$ are the historical maximum and minimum NDVI of the same period, respectively, using long-term time-series NDVI datasets. Considering that the minimum crop NDVI may be contaminated by clouds or non-vegetation pixels, an empirical minimum vegetation NDVI value (0.15) was introduced to calculate ${{NDVI}}_{\min {\_h}}$ using the following equation:
  $${{NDVI}}_{\min {\rm{\_}}h}=\max (0.15,{{NDVI}}_{\min {\rm{\_}}h0})$$
  where ${{NDVI}}_{\min {\_h}0}$ is the original minimum NDVI of the study period from the time-series NDVI dataset. The VCIx values ranged from zero to 1. A value of 0 indicates that the vegetation condition corresponded to the worst level in recent decades, 1 indicates that the vegetation condition corresponded to the best level in recent decades, and a value greater than 1 indicates that the vegetation condition during the current observation period exceeded the historical optimum level.
  These indicators reflect the crop growth conditions and area. To process these data, we used crop phenology data to divide the crop condition indicators into time windows that served as feature inputs to the model.
11. **GLASS Remote Sensing Data Products**. In this study, we used GLASS remote sensing data products^40^ including net primary productivity (NPP) and leaf area index (LAI). The GLASS products are derived from multiple satellite observations and provide consistent and reliable information on vegetation dynamics. NPP represents the net amount of carbon assimilated by vegetation through photosynthesis, whereas LAI refers to the total one-sided area of leaf tissue per unit ground surface area. These data comprehensively and accurately reflect the growth status of vegetation and the intensity of photosynthetic activity.

NPP is provided as annual data, whereas LAI is provided as time-series data with a frequency of every nine days. These MODIS-derived products offer global coverage with multiple spatial resolution options. The NPP dataset covers the period from 2000 to 2020, while the LAI dataset spans from 2000 to 2021. For our study, we specifically utilized data from 2010 to 2020, aligning with our research timeframe. To process the LAI data, we divided the crop phenology data into time windows and calculated the maximum, minimum, standard deviation, and sum of the data within each time window, which served as the feature inputs for the model.

### Data-driven approach

We employed a comprehensive data-driven approach to generate the GGCP10 dataset. The methodology centers on a series of adaptively trained machine learning models, tailored to capture the unique characteristics of crop production in different agroecological zones. The approach encompasses four key stages: harvested area calculation, multi-source feature extraction, data-driven model training, and production estimation.

1. **Harvested Area Calculation**

Harvested area is a crucial foundation for estimating crop production. Harvested area data contain rich agricultural production information, such as multiple cropping indices and rotation patterns, which are key factors in assessing regional agricultural planting structure and land use intensity and have important research value. However, production is the product of harvested area and yield, and estimating harvested area separately helps us to better understand the relative contributions of these two factors to production changes and deepen our understanding of the production composition mechanism.

This study proposes a dynamic harvested area calculation algorithm at an annual scale to accurately estimate the harvested area of each grid cell in the target year. The key innovation of this method lies in the introduction of seasonal CALF data to reflect dynamic changes in cropland utilization intensity, which are deeply integrated with statistical and spatial distribution data from the reference year to estimate the harvested area at the pixel scale for the target year. The CALF data reflect the agricultural planting intensity of each grid cell in different seasons, characterizing the spatial and temporal differences in agricultural planting across different regions. In contrast, the traditional fixed-proportion method^14^ calculates the harvested area of each grid cell in the target year based on statistical and gridded harvested area data from a reference year using proportional allocation, ignoring the changes in cropping conditions between different grid cells within a region.

Specifically, to calculate a country’s harvested area in the target year, the method is divided into three steps: (1) Cropping area calculation at the national scale. Based on phenological data, seasonal CALF data, and the spatial distribution of the harvested area in the reference year, the crop-specific cropping area of each country in different seasons was estimated at the pixel scale, ultimately obtaining the annual crop-specific cropping area. (2) Harvested area allocation at the AEZ scale. By analyzing the interannual changes in the cropping area within each AEZ, this step allocates national-level harvested area statistics to each AEZ to obtain the total harvested area for each subregion. (3) Harvested area mapping at the pixel scale. Within each AEZ, considering the density and interannual changes in the cropping area, this step constructs a spatial allocation model at the pixel scale to further decompose the total harvested area of each AEZ into each pixel, ultimately generating the gridded harvested area data.

**Step 1: Gridded cropping area calculation for the target year**

**Input data:**

1. Phenological information for country c
2. Harvested area of country c in the reference year (2015, from the GAEZ dataset)
3. CALF data for country c in four growing seasons of the target year

**Output data:**

1. Crop-specific cropping area of each pixel in country c for the target year

**① Temporal scale conversion of phenological data**. According to the 10-day resolution phenological information, aggregate it into 4 growing seasons to obtain phenological information at the seasonal time resolution. Let ${C}_{j,t}$ denote the phenological value (0 or 1) of crop j in growing season t, where 0 indicates no planting of the crop in that season and 1 indicates planting.

**② Calculate the total cropping area for each season**. Using the CALF data of the four growing seasons and the cropland mask data, calculate the total cropping area of each grid cell in each season by multiplying the cropland area by the cropland planting ratio. Let ${{CA}}_{i,t}$ denote the total cropping area of grid cell i in season t.

$${{CA}}_{i,t}={CroplandAre}{a}_{i}\times {CAL}{F}_{i,t}$$

where ${CroplandAre}{a}_{i}$ represents the cropland area of grid cell i, and ${{CALF}}_{i,t}$ represents the cropland planting ratio of grid cell i in season t.

**③ Allocate cropping area based on phenological information**. For each growing season t, determine the set of crop types ${S}_{t}$ according to the phenological information. Then, for crop j in grid cell i, calculate its cropping area ${{CA}}_{i,j,t}$ in season t using the following formula:

$${{CA}}_{i,j,t}={{CA}}_{i,t}\times \frac{{{HA}}_{i,j}}{{\sum }_{k\in {S}_{t}}{{HA}}_{i,k}}\times {C}_{j,t}$$

where ${{CA}}_{i,j,t}$ represents the cropping area of crop j in grid cell i in season t, ${C}_{j,t}$ represents the phenological value (0 or 1) of crop j in season t, with 0 indicating no planting and 1 indicating planting, ${{CA}}_{i,t}$ represents the total cropping area of grid cell i in season t, ${{HA}}_{i,j}$ represents the harvested area of crop j in grid cell i in the reference year (i.e., 2015, from the GAEZ + 2015 dataset), and ${S}_{t}$ represents the set of all planted crops in season t.

**④ Calculate the annual cropping area**. By summing the cropping areas of each season, obtain the annual cropping area $C{A}_{i,j}$ of crop j in grid cell i:

$$C{A}_{i,j}=\mathop{\sum }\limits_{t=1}^{4}C{A}_{i,j,t}$$

**Step 2: Harvested area allocation at the AEZ scale**

**Input data:**

1. Crop-specific cropping area of each grid cell in the target year (output from Stage 1)
2. Gridded harvested area data in the reference year (2015) (from the GAEZ + 2015 dataset)
3. Crop-specific cropping area of each grid cell in the reference year
4. Statistical data of crop-specific harvested area in the target year (from the FAO database)
5. Agro-ecological zoning (AEZ) data

**Output data:**

1. Statistical values of harvested area for each AEZ within country c

**① Calculate the initial harvested area proportion of each AEZ**. For crop j, AEZ k, and country c, calculate the initial harvested area proportion ${w}_{j,k,c}^{{init}}$ of AEZ k within country c in the reference year:

$${w}_{j,k,c}^{{init}}=\frac{\sum _{m\in k}H{A}_{m,j,k}^{{ref}}}{\sum _{n\in c}H{A}_{n,j,c}^{{ref}}}$$

where ${\sum }_{m\in k}H{A}_{m,j,k}^{{ref}}$ represents the sum of harvested areas of crop j in all grid cells within AEZ k in the reference year, and ${\sum }_{n\in c}H{A}_{n,j,c}^{{ref}}$ represents the sum of harvested areas of crop j in all grid cells within country c in the reference year.

**② Adjust the harvested area proportion of each AEZ**. According to the ratio of crop-specific cropping area within each AEZ between the target year and the reference year, adjust the harvested area proportion of each AEZ to obtain the harvested area proportion ${w}_{j,k,c}$ of each AEZ in the target year:

$${w}_{j,k,c}={w}_{j,k,c}^{{init}}\times \frac{\sum _{m\in k}C{A}_{m,j,k}^{{tar}}/\sum _{m\in k}C{A}_{m,j,k}^{{ref}}}{\sum _{n\in c}C{A}_{n,j,c}^{{tar}}/\sum _{n\in c}C{A}_{n,j,c}^{{ref}}}$$

where ${\sum }_{m\in k}C{A}_{m,j,k}^{{tar}}$ and ${\sum }_{m\in k}C{A}_{m,j,k}^{{ref}}$ represent the sum of cropping areas of crop j in all grid cells within AEZ k in the target year and the reference year, respectively, and ${\sum }_{n\in c}C{A}_{n,j,c}^{{tar}}$ and ${\sum }_{n\in c}C{A}_{n,j,c}^{{ref}}$ represent the sum of cropping areas of crop j in all grid cells within country c in the target year and the reference year, respectively.

**③ Calculate the total harvested area of each AEZ**. Using the FAO statistical data of national crop-specific harvested area and the harvested area proportion of each AEZ, calculate the total harvested area $H{A}_{j,k,c}^{{tar}}$ of each AEZ for crop j in the target year:

$$H{A}_{j,k,c}^{{tar}}=H{A}_{j,c}^{{tar}}\times {w}_{j,k,c}$$

where $H{A}_{j,c}^{{tar}}$ represents the total harvested area of crop j within country c in the target year, which can be directly obtained from the FAO database.

**Step 3: Calculate the harvested area of grid cells within each AEZ**

**Input data:**

1. Crop-specific cropping area of each grid cell in the target year (output from Stage 1)
2. Gridded harvested area data in the reference year (2015) (from the GAEZ + 2015 dataset)
3. Crop-specific cropping area of each grid cell in the reference year (output from Stage 1)
4. Statistical values of harvested area for each AEZ within country c (output from Stage 2)

**Output data:**

1. Gridded harvested area data of crop j in the target year

**① Calculate the spatial weight of grid cells within each AEZ**. For each AEZ, calculate the proportion of the harvested area of each grid cell in the total harvested area of the AEZ in the reference year as the spatial weight within the AEZ. For grid cell i, crop j, and AEZ k, the spatial weight ${w}_{i,j,k}^{{ref}}$ is calculated as:

$${w}_{i,j,k}^{{ref}}=\frac{H{A}_{i,j,k}^{{ref}}}{\sum _{m\in k}H{A}_{m,j,k}^{{ref}}}$$

where $H{A}_{i,j,k}^{{ref}}$ represents the harvested area of crop j in grid cell i within AEZ k in the reference year, and ${\sum }_{m\in k}H{A}_{m,j,k}^{{ref}}$ represents the sum of harvested areas of crop j in all grid cells within AEZ k in the reference year.

**② Adjust the spatial weight of grid cells within each AEZ**. Using the cropping area change information calculated in the last step, adjust the spatial weight within each AEZ in the reference year to obtain the spatial weight ${w}_{i,j,k}^{{tar}}$ within each AEZ in the target year:

$${w}_{i,j,k}^{{tar}}={w}_{i,j,k}^{{ref}}\times \frac{{r}_{i,j,k}}{{r}_{j,k}}$$

where ${r}_{i,j,k}$ represents the change rate of cropping area of crop j in grid cell i within AEZ k, and ${r}_{j,k}$ represents the change rate of total cropping area of crop j within AEZ k. Their calculation formulas are:

$${r}_{i,j,k}=\frac{C{A}_{i,j,k}^{{tar}}}{C{A}_{i,j,k}^{{ref}}}$$

$${r}_{j,k}=\frac{\sum _{m\in k}C{A}_{m,j,k}^{{tar}}}{\sum _{m\in k}C{A}_{m,j,k}^{{ref}}}$$

where $C{A}_{i,j,k}^{{tar}}$ and $C{A}_{i,j,k}^{{ref}}$ represent the cropping area of crop j in grid cell i within AEZ k in the target year and the reference year, respectively.

**③ Calculate the harvested area of grid cells within each AEZ**. Using the total harvested area $H{A}_{j,k,c}^{{tar}}$ of each AEZ calculated in ③ of Step 2 and the spatial weight ${w}_{i,j,k}^{{tar}}$ within each AEZ, calculate the harvested area $H{A}_{i,j,k}^{{tar}}$ of crop j in grid cell i within AEZ k in the target year:

$$H{A}_{i,j,k}^{{tar}}=H{A}_{j,k,c}^{{tar}}\times {w}_{i,j,k}^{{tar}}$$

**④ Data consistency validation**. At the national scale, aggregate the gridded harvested area data and compare it with the FAO statistical data. If there is a discrepancy between the two, uniformly adjust the gridded data according to the discrepancy ratio to make its total equal to the statistical data.

Using this three-step algorithm, we calculated gridded harvested areas for the target year. This method comprehensively utilizes multisource data, including CALF data, spatial distribution data from the reference year, agricultural statistical data, and crop phenological information. Compared with traditional methods, this method provides significant improvements in the spatiotemporal continuity of data sources. Second, in terms of the calculation strategy, this method adopts a subregional and multistage approach. By dividing the estimation process into three scales–national, AEZ, and pixel–and conducting estimations for cropping and harvested areas separately, the spatiotemporal distribution pattern of the harvested area in the target year can be accurately characterized, providing important data support for subsequent production estimation.

(2) **Multi-Source Feature Extraction**

To comprehensively characterize the key factors influencing crop growth and development, we considered natural factors such as meteorology, soil, and terrain, as well as human management factors such as irrigation and cultivation, when selecting features. At the same time, to capture the dynamic process of crop growth, we fully utilized multi-source time-series data and extracted environmental condition information at different growth stages. Therefore, we adopted a phenology-based feature extraction method to transform the original spatiotemporal data into structured feature vectors to form a multidimensional representation of the crop growth conditions.

Specifically, we extracted features from multi-source data using the following steps.

**Step 1: Determine key growth periods**. We first determined the time ranges of the main growth stages (from sowing to harvest) for each crop type, based on crop phenological information. The crop phenological data had an original temporal resolution of 10 d. We processed the phenological data to match the temporal resolutions of the different data sources. For example, for input data with a quarterly resolution (e.g., four quarters in one year), we aggregated the original 10 d phenological data and sampled them into four quarters, thereby achieving the temporal synchronization of phenological information with other data.

**Step 2: Time-series features extraction**. After determining the key growth periods, we extracted multi-source time-series data, such as remote sensing, meteorological, and soil data, within the corresponding time windows according to the phenological rhythm of each crop. For continuous variables (e.g., precipitation and temperature), we calculated a series of statistical features within key growth periods, including:

1. Cumulative amount: reflecting the overall resource input or environmental pressure during the growth period
2. Mean value: characterizing the average condition during the growth period
3. Maximum and minimum values: reflecting the extreme conditions during the growth period
4. Standard deviation: measuring the variability during the growth period

Using the above statistical features, we can comprehensively characterize the overall characteristics and dynamic changes in environmental factors during each growth period, providing rich information dimensions for subsequent production estimation.

**Step 3:** Process categorical variables. We used One-Hot Encoding to transform the categorical variables (e.g., irrigation type). One-Hot Encoding is a commonly used method for processing categorical variables. It converts each category into a binary vector, allowing categorical variables to be directly input into numerical models. For example, for the irrigation type (irrigated, rainfed, or unknown), One-Hot Encoding generates three new binary variables, indicating whether the sample belongs to the corresponding irrigation type. Through One-Hot Encoding, we retained the information of categorical attributes while keeping them consistent in form with numerical variables, facilitating subsequent modeling and analysis.

**Step 4:** Construct a structured feature set. Through statistical feature extraction of continuous variables and One-Hot Encoding of categorical variables, we obtained a structured feature set (Table 2) covering the key spatiotemporal dimensions of crop growth. This feature set contained environmental and management factors from multiple data sources and various data types, with each factor integrated into one or more feature dimensions according to its spatiotemporal attributes. For example, precipitation data were extracted into four feature dimensions (cumulative amount, mean value, maximum value, and minimum value), whereas irrigation type data were encoded into three feature dimensions (irrigated, rainfed, and unknown). Through this multi-dimensional and multiscale feature representation, we constructed a comprehensive and compact crop growth factor library, laying an important foundation for subsequent machine learning modeling.

**Table 2:** Input data and extracted features used for the crop production estimation model.

Input data	Observations per year	Name for features	Name for Sub features	Number of features
Harvested area	1	HA	HA	1
Maximum vegetation condition index	4	VCIx	VCIx_1, VCIx_2, VCIx_3, VCIx_4	4
Cropped arable land fraction	4	CALF	CALF_1, CALF_2, CALF_3, CALF_4	4
Cumulative potential biomass	4	BIOMASS	BIOMASS_1, BIOMASS_2, BIOMASS_3, BIOMASS_4	4
Cumulative precipitation	36	RAIN	RAIN_Min, RAIN_Max, RAIN_Sum, RAIN_Std	4
Photosynthetically active	36	PAR	PAR_Min, PAR_Max, PAR_Sum, PAR_Std	4
Average air temperature	36	TEMP	TEMP_Min, TEMP_Max, TEMP_Mean, TEMP_Std	4
Net primary productivity	1	NPP	NPP	1
Leaf area index	46	LAI	LAI_Min, LAI_Max, LAI_Sum, LAI_Std	4
Location data	Static	Location	Location_gx, Location_gy, Location_gz	3
Terrain data	Static	Terrain	Terrain_Elevation, Terrain_Variation	2
Soil texture data	Static	Soil	Soil_Clay, Soil_Sand, Soil_Silt	3
Irrigation Type	Static	Irrigation Type	Irrigation Type_1, Irrigation Type_2, Irrigation Type_3	3
Total				41

Compared with traditional feature construction methods based on static indicators or a single timescale, the phenology-driven time-series feature extraction method adopted in this study has notable advantages. By organically combining the temporal dynamics and spatial differentiation of environmental factors, this method can comprehensively and accurately characterize the multidimensional influencing mechanisms of crop growth and development processes. For example, calculating the cumulative precipitation and average temperature during the growing season can reflect the water and heat conditions during the crop growth period, thereby affecting biomass accumulation and yield. By extracting remote-sensing vegetation indices (such as VCIx) at different growth stages, spatiotemporal changes in crop growth can be dynamically monitored, grasping the key stages and regional differences in yield formation.

Moreover, the feature vectors constructed in this study have clear physical meaning and ecological basis. For example, the absorption and utilization of photosynthetically active radiation (PAR) during crop growth are fundamental driving forces for yield. Therefore, incorporating the temporal features of PAR can help us understand the potential causes of yield variation from a mechanistic perspective. Similarly, environmental factors such as soil texture and terrain features influence crop growth by regulating the availability of water and nutrients. Including the spatial features of these factors contributes to explaining the regional differentiation patterns of yield. Through the above mechanistic feature selection, the feature vectors we constructed can not only support efficient production prediction but also uncover key factors or limiting factors of regional crop production.

(3) **Data-Driven Model Training**

Crop production is a complex biophysical process influenced by numerous environmental factors in a nonlinear manner. Traditional parametric methods often struggle to capture these nonlinearities and multiscale effects fully, limiting the accuracy and reliability of production estimation. In contrast, machine learning methods have unique advantages in characterizing the response patterns of production to changes in environmental factors and exploring the key factors influencing production formation. By constructing complex nonlinear models, machine learning methods can better reveal the inherent mechanisms of the production formation process, thereby providing new ideas for precise production prediction. This data-driven modeling paradigm has been successfully applied in the field of agricultural remote sensing^21,41–44^. Furthermore, the complex relationships between production and environmental factors exhibit significant spatial regional differentiation, making regional modeling extremely necessary.

To uncover the intrinsic relationships between crop production and multi-source influencing factors, this study adopted a data-driven modeling paradigm and employed an agroecological zoning modeling strategy. We constructed a set of targeted production estimation models for each crop type and agro-ecological zone (AEZ) to improve the accuracy and reliability of production mapping. The model uses the harvested area (HA) and multi-source indicators (XI) at the grid scale as inputs, and estimates the crop production (P) of the corresponding grid cell. Its basic form can be expressed as:

$${P}_{i,j}=f\left({{HA}}_{i,j},{{XI}}_{i,j}\right)$$

where the variables ${P}_{i,j}$, ${{HA}}_{i,j}$ and ${{XI}}_{i,j}$ respectively represent production, harvested area, and various multi-source indicators in grid cell *i* of crop type *j*. *f* is a machine-learning model customized for each AEZ and crop type to capture the unique relationship between production, harvested area, and indicators.

Considering the good performance and robustness of ensemble learning models, this study selected Random Forest^45^, XGBoost^46^, and CatBoost^47^ as candidate models for geospatial modeling of crop production. Among them, Random Forest is one of the most commonly used algorithms in ensemble learning; XGBoost has achieved excellent results in various data mining competitions and has been proven to have significant advantages in processing high-dimensional and nonlinear relationship data; CatBoost has shown outstanding performance in multiple data science competitions, and is considered a powerful tool for handling mixed data.

For each specific crop type, data-driven models were built independently for each AEZ based on its geographical subdivision. In other words, one model was trained for each crop in each AEZ. The model training and optimization were divided into two steps: (1) Best model selection and (2) Model training.

**Step 1: Best model selection**. In the first step, a nested cross-validation strategy was employed to perform hyperparameter optimization and performance evaluation of the three candidate models. Specifically,

1. First, samples from the reference year were divided into a training set (90%) and a test set (10%).
2. In the training set, five-fold cross-validation was used to perform grid search optimization of the model hyperparameters, such as the number of trees and maximum depth of the Random Forest, learning rate, and number of trees of XGBoost and CatBoost, to find the optimal hyperparameter configuration for each model.
3. The models were retrained using the optimized hyperparameters and evaluated for their predictive performance on a test set. The model performance evaluation metric was the coefficient of determination (R²), which measures the model’s ability to explain yield variation.
4. The model with the highest R² value in the test set was selected as the optimal model for AEZ.

It should be emphasized that the above hyperparameter optimization and model evaluation processes were performed independently within each AEZ to obtain the optimal model adapted to the characteristics of different regions. While this strategy helps improve the model’s ability to characterize regional production variation patterns and enhances the accuracy of regional production estimations, it’s worth noting that for AEZs with very small sample sizes, the robustness of the results may be somewhat reduced. The optimal model selection results are presented in the Performance Evaluation of Data-Driven Models of Technical Validation.

**Step 2: Model training**. In the second stage, considering the limited data resources and importance of these data for prediction, we made full use of all the data from the reference year. Therefore, the optimal model and corresponding optimal parameters selected in the first stage were combined with all reference year data to retrain the model and improve its stability and prediction accuracy, thus obtaining the production prediction model for each AEZ.

In summary, this study adopted a data-driven modeling strategy that fully utilized agroecological zoning information to construct a set of machine learning models for regionalized crop production estimation. Model hyperparameter optimization and performance evaluation were achieved through nested cross-validation, and the final predictive models were trained based on all reference year data. This method not only improves production estimation accuracy but also characterizes the regional heterogeneity of production response mechanisms.

(4) **Production Estimation**

Production calculation involves the conversion of multisource feature data into gridded production estimates through the application of spatial production estimation models. This process was performed for each crop type and agro-ecological zone (AEZ) to generate initial production estimates, which were then calibrated using national-level statistics to ensure consistency. The detailed steps are as follows.

**Step 1: Feature data preparation**. For each crop type j and AEZ k in country c, the corresponding feature data (as described in Table 2) were extracted and organized into a structured dataset. This dataset included various indicators related to crop growth and production. The feature dataset serves as the input for the subsequent estimation of spatial production.

**Step 2: Spatial production estimation**. Based on the feature dataset prepared in Step 1, the trained spatial production estimation model for crop type j and AEZ k was applied to predict the production of each grid cell i within the AEZ. This process is expressed as follows:

$${P}_{i,j,k}^{{init}}={f}_{j,k}\left({X}_{i,j,k},H{A}_{i,j,k}\right)$$

where ${P}_{i,j,k}^{{init}}$ represents the initial production estimate of grid cell i for crop type j in AEZ k; ${f}_{j,k}$ denotes the trained production estimation model for crop type j and AEZ k; ${X}_{i,j,k}$ represents the feature vector of grid cell i for crop type j in AEZ k; and $H{A}_{i,j,k}$ represents the estimated harvested area of grid cell i for crop type j in AEZ k.

By applying the spatial production estimation model to all grid cells within each AEZ, we obtain a gridded dataset of initial production estimates ${P}_{i,j,k}^{{init}}$ for each crop type j in Country c.

**Step 3: Production calibration**. While spatial production estimation models provide a robust foundation for predicting crop production at the grid level, model-derived estimates may not always be perfectly consistent with established agricultural statistics because of various factors such as model limitations and data anomalies. To address this issue and ensure the consistency of our dataset with official statistics, we performed a calibration process using national-level production data from the FAO.

For each crop type j in country c, the calibration process involved the following steps:

1. Calculate the total initial production estimate for country c by summing the initial production estimates of all grid cells within the country.
  $${P}_{j,c}^{{init}}=\sum _{k\in c}\sum _{i\in k}{P}_{i,j,k}^{{init}}$$
2. Retrieve the official national production statistics ${P}_{j,c}^{{FAO}}$ for crop type j in country c from the FAO database.
3. Calculate the calibration coefficient ${{\rm{\alpha }}}_{j,c}$ by dividing the FAO national production statistics by the total initial production estimate:
  $${{\rm{\alpha }}}_{j,c}=\frac{{P}_{j,c}^{{FAO}}}{{P}_{j,c}^{{init}}}$$
4. Apply the calibration coefficient to adjust the initial production estimate of each grid cell, obtaining the final calibrated production estimate:

$${P}_{i,j,k}^{{cal}}={{\rm{\alpha }}}_{j,c}\times {P}_{i,j,k}^{{init}}$$

By performing this calibration process for each crop type and country, we ensured that the sum of the calibrated grid-level production estimates matched the FAO national statistics, thereby providing a consistent and harmonized dataset for agricultural production analysis.

It is important to note that although the calibration process ensures consistency with official statistics at the national level, it does not necessarily imply absolute accuracy at the grid scale. Official statistics may contain uncertainties and the calibration process inherits these uncertainties. However, maintaining consistency with official statistics provides a unified benchmark for comparative analysis of agricultural production across different regions and years, thereby enhancing the reliability and utility of the GGCP10 dataset.

## Data Records

### Dataset structure and accessibility

The GGCP10 dataset is publicly available through the Harvard Dataverse repository^24^ and can be accessed at 10.7910/DVN/G1HBNK. This dataset provides annual crop production information for four major crops (maize, wheat, rice, and soybean) from 2010 to 2020. The dataset covers the entire globe and is organized in a gridded format with a spatial resolution of 10 km. The data are stored in GeoTIFF format, which is widely compatible with various geospatial analysis software.

Key characteristics of the GGCP10 dataset:

1. **Spatial coverage:** Global (180°W to 180°E, 90°N to 90°S)
2. **Spatial resolution:** 0.08333333 degrees (approximately 10 km at the equator)
3. **Temporal coverage:** 2010 to 2020 (annual)
4. **Coordinate Reference System:** EPSG:4326 - WGS 84
5. **File format:** GeoTIFF
6. **Data type:** 32-bit floating point
7. **Number of bands:** 1 (single band per file)
8. **Unit:** Kilotons per 100 square kilometers
9. **No data value:** Oceans, open-water, and Antarctica in the GeoTIFF files have the no-data value of -9999.

The dataset is organized into separate files for each crop type and year, resulting in a total of 44 GeoTIFF files (11 years × 4 crop types). Each pixel value represents the estimated crop production for the corresponding 10 km × 10 km grid cell in kilotons.

File naming convention: GGCP10_Production_[Year]_[Crop].tif. Where [Year] ranges from 2010 to 2020, and [Crop] is one of {Maize, Wheat, Rice, Soybean}.

Example: GGCP10_Production_2020_Maize.tif represents the global maize production for the year 2020.

Users can access and analyze this dataset using common GIS software or programming languages with geospatial libraries such as GDAL, rasterio (Python), or terra (R).

### Spatial features of crop production in GGCP10

**Fig. 3:**
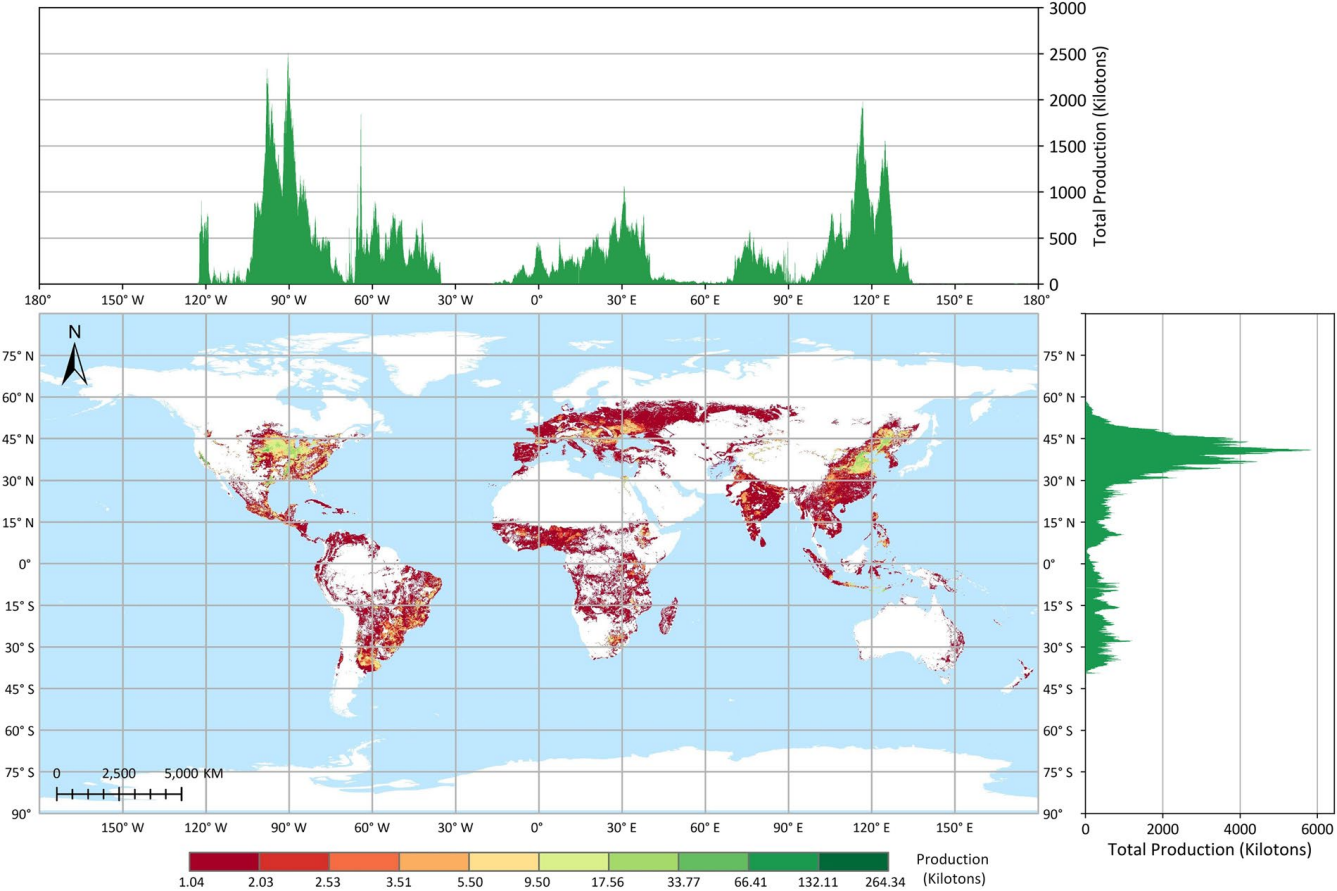
Global distribution of maize production in 2020 based on the GGCP10 dataset. High-production regions are predominantly located in the U.S. Corn Belt, northeastern and northern China, southern Brazil, the Pampas of Argentina, and the northwestern Black Sea regions of Ukraine and Romania. Latitudinally, production is concentrated between 30°N and 45°N. Longitudinally, peak production is observed near 90°W, 60°W, 30°E, and 120°E, corresponding to major agricultural zones in North and South America, Europe, and East Asia.

**Fig. 4:**
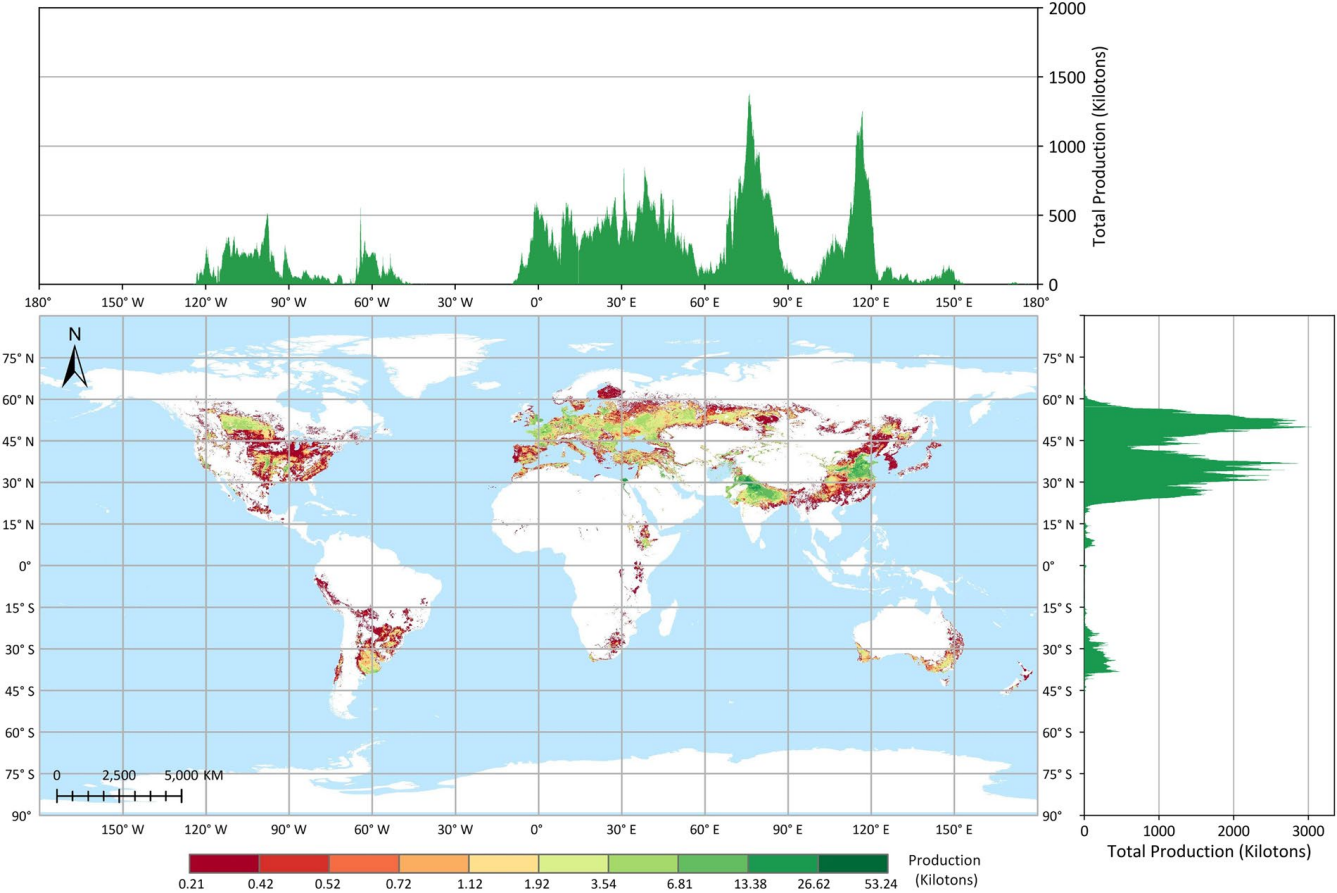
Global distribution of wheat production in 2020 based on the GGCP10 dataset. Major wheat-producing regions include northern China, northern India, Europe, the Nile River Delta, southern Canada, and Argentina. Latitudinally, production is concentrated between 60°N and 20°N, encompassing the primary wheat-growing belts. Longitudinally, production peaks are observed in three main zones: 0°–50°E (Europe and Western Asia), 70°–85°E (South Asia), and 110°–120°E (East Asia), reflecting the global diversity of wheat cultivation areas.

**Fig. 5:**
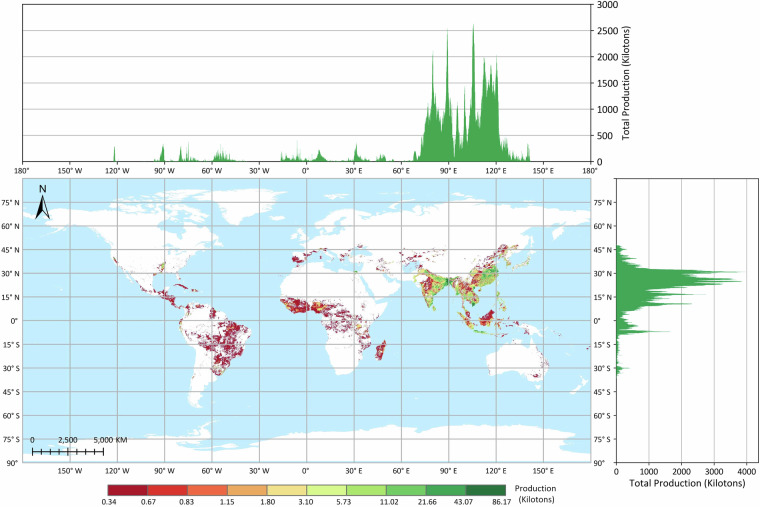
Global distribution of rice production in 2020 based on the GGCP10 dataset. Regions with high rice production are predominantly located in South China, Southeast Asia, and South Asia. Latitudinally, production is concentrated between 35°N and 10°N, encompassing the primary rice-growing tropical and subtropical zones. Longitudinally, the majority of production is observed between 75°E and 120°E, corresponding to the rice-intensive areas of South and East Asia.

**Fig. 6:**
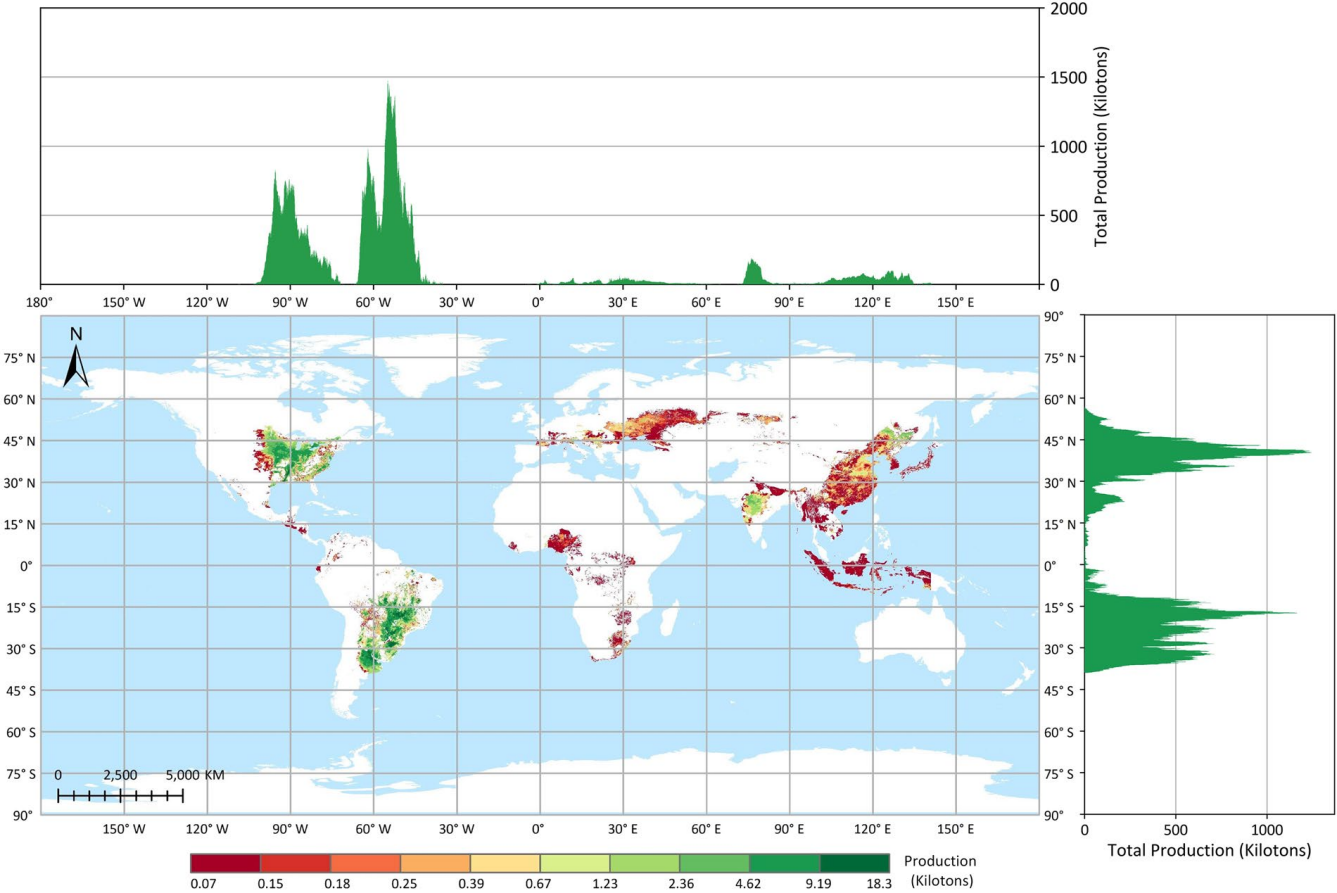
Global distribution of soybean production in 2020 based on the GGCP10 dataset. High-production areas are primarily concentrated in the eastern Great Plains of the United States, southern Brazil, and northern Argentina, with additional significant production zones in Northeast China and central India. The latitudinal distribution exhibits a bimodal pattern, with production peaks between 15°S and 35°S in the Southern Hemisphere and between 30°N and 50°N in the Northern Hemisphere. Longitudinally, production also shows a bimodal distribution, with major peaks around 90°W and 65°W, corresponding to the primary soybean-growing regions in North and South America.

The GGCP10 dataset provides high-resolution gridded crop production data at a spatial resolution of 10 km × 10 km, covering a time span of 11 years from 2010 to 2020. The dataset includes four major crop types: maize, wheat, rice, and soybean. The pixel values in the GGCP10 dataset represent the estimated crop production within each grid cell, with the unit being kilotons. To illustrate the spatial patterns of crop production, we selected the data from the year 2020 for visualization and analysis, as shown in Figs. 3, 4, 5 and 6.

## Technical Validation

We conducted a comprehensive two-part validation of the GGCP10 dataset to assess its reliability and accuracy. First, we evaluated the performance of our crop production estimation models across different agroecological zones, analyzing both model selection results and regional variations in model accuracy. Second, we performed extensive comparisons between GGCP10 and multiple reference datasets, including global gridded products, regional gridded datasets, and subnational statistics from diverse geographical contexts spanning 43 countries.

### Performance evaluation of data-driven models

**Fig. 7:**
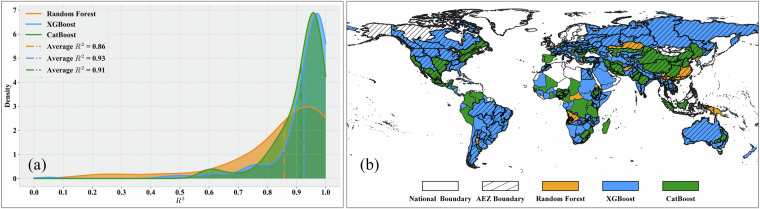
Model selection results for maize production estimation. (**a**) Gaussian kernel density of R² values; (**b**) Global spatial distribution of optimal models.
  For wheat, a total of 237 regional models were trained, including 138 XGBoost models, 82 CatBoost models, and 17 RF models. As shown in Fig. 8(a), CatBoost achieved the highest R² values among the three models, with an average of 0.93. The XGBoost models also exhibited high R² values, with an average of 0.92 and a more concentrated accuracy distribution. The average R² value of RF models is 0.90. From the spatial distribution perspective (Fig. 8(b)), CatBoost shows better performance in the main wheat-planting areas of North America and Asia, while XGBoost dominates in the main planting areas of Europe and South America. Wheat planting areas in Africa and Oceania are relatively few, and the model selection results are more dispersed.

1. **Results of model selection**. In the first stage of data-driven model training, we selected the optimal model for each modeling unit through cross-validation. In this section, we use Gaussian kernel probability density plots of R² to illustrate the accuracy distribution of the three types of machine learning models among these optimal models. The horizontal coordinate represents the R² value, and the vertical coordinate shows the Gaussian kernel probability density of R². Additionally, we spatially display the type of optimal model corresponding to each modeling unit, using different colors to denote the optimal machine learning model for each unit.
  For maize, a total of 303 regional models were trained, comprising 199 XGBoost models, 79 CatBoost models, and 25 RF models. It is important to note that the number of models constructed equals the number of modeling units, as modeling is only performed for units with maize production. Thus, for maize, we selected 303 modeling units globally. As shown in Fig. 7(a), from the accuracy distribution perspective, XGBoost and CatBoost exhibit higher accuracy, with XGBoost models having an average R² of 0.93, CatBoost models an average R² of 0.91, and RF models an average R² of 0.86. Figure 7(b) illustrates the spatial distribution perspective, revealing that the XGBoost model performs best in most regions of North America, South America, Europe, Asia, and parts of Africa, while Random Forest and CatBoost dominate in a few regions of Africa, Europe, and Asia.

**Fig. 8:**
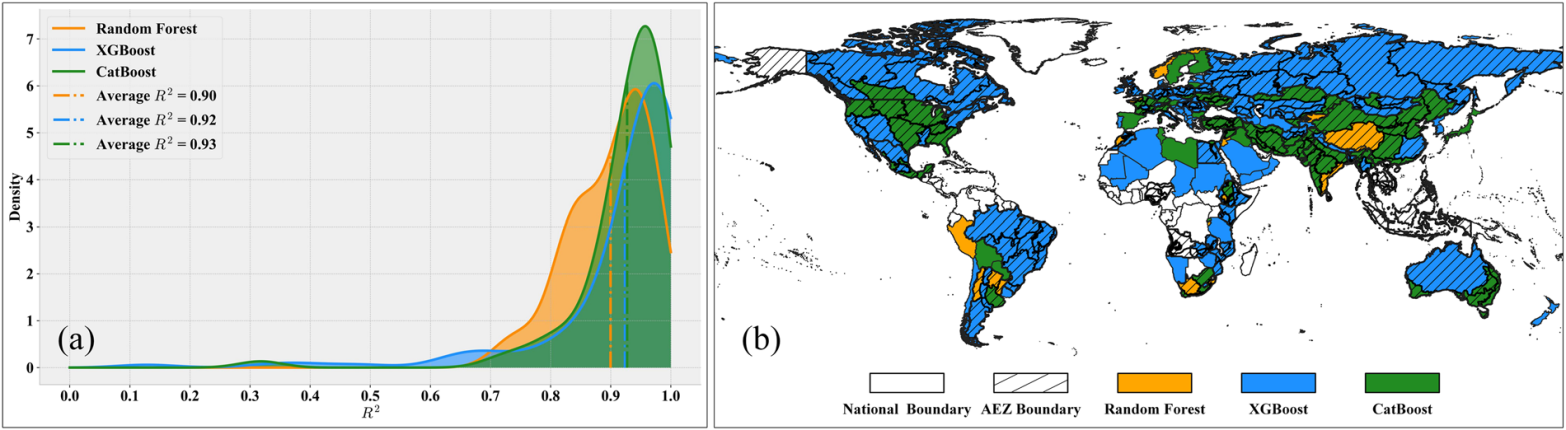
Model selection results for wheat production estimation. (**a**) Gaussian kernel density of R² values; (**b**) Global spatial distribution of optimal models.
  For rice, out of 202 models, XGBoost, CatBoost, and RF models accounted for 145, 37, and 20 models, respectively. As shown in Fig. 9(a) and Fig. 9(b), XGBoost accounts for the majority and has the highest average R² of 0.94. However, for the major rice-producing regions globally (South Asia and Southeast Asia), CatBoost is the optimal model for most regions, with an average R² of 0.90, slightly lower than XGBoost. This indicates that for these major rice-producing regions, the modeling difficulty is relatively high due to their complex cropping systems, diverse environmental conditions, and relatively fragmented rice paddies. The number of random forest models is small, with an average R² of 0.90.

**Fig. 9:**
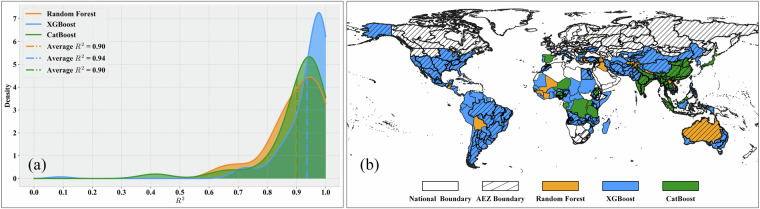
Model selection results for rice production estimation. (**a**) Gaussian kernel density of R² values; (**b**) Global spatial distribution of optimal models.
  For soybean, out of the 155 models, 84 were XGBoost, 54 were CatBoost, and 17 were RF. As shown in Fig. 10(a) and Fig. 10(b), CatBoost dominates the main producing areas in South America, North America, and South Asia, with an average R² value of 0.88, while XGBoost is mainly distributed in Europe, southern Africa, and other regions, with an average R² value of 0.86. Although the average R² of random forest is not low, the variation range is large, and the corresponding regions are not the main soybean-producing areas

**Fig. 10:**
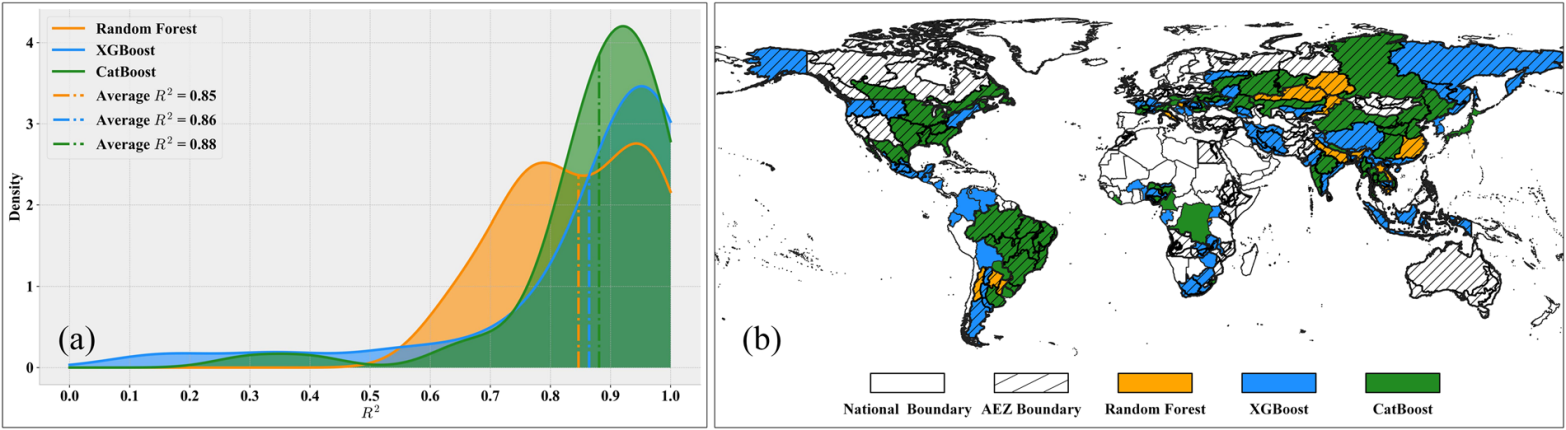
Model selection results for soybean production estimation. (**a**) Gaussian kernel density of R² values; (**b**) Global spatial distribution of optimal models.
  Overall, the probability density plots indicate that for all four crops, the R² values of XGBoost, CatBoost, and RF are high, with narrow ranges, demonstrating good model accuracy and stability. For all four crops, the number of XGBoost models is significantly higher than that of CatBoost or RF, suggesting that XGBoost exhibits the best model performance in most regions, consistent with the conclusions from our previous study^21^. From the spatial distribution perspective, there is a certain degree of geographical differentiation in the applicability of different models. For example, XGBoost has more advantageous regions in Africa, Asia, and Europe, while CatBoost performs prominently in some parts of the Americas and Oceania. This differentiation pattern may be related to differences in regional agricultural planting systems and ecological environmental conditions.
  These results demonstrate the necessity of adaptively selecting optimal models and parameters to train relatively accurate models for various crops and regions. In-depth analysis of the correlation between model selection results and regional characteristics (such as climate, soil, topography, etc.) will help understand the geographical differentiation patterns of model applicability and provide references for optimizing models and algorithms according to local conditions.
2. **Evaluation of Model Performance in Different Regions**. The previous section introduced the results of optimal model selection in different regions worldwide, demonstrating the differences in model applicability across regions. Building on this foundation, this section further evaluates model performance at the continental scale, aiming to reveal regional variation patterns in crop production estimation model performance and explore the relationship between production estimation difficulty and regional agricultural characteristics. To this end, we grouped regional models according to continents and used violin plots to display the distribution characteristics of model performance (R²) for four crops in each continent, as shown in Fig. 11. Violin plots show the overall distribution of model performance and present statistical information such as maximum, minimum, median, and quartiles of model performance in different regions, facilitating comparative analysis between regions.

**Fig. 11:**
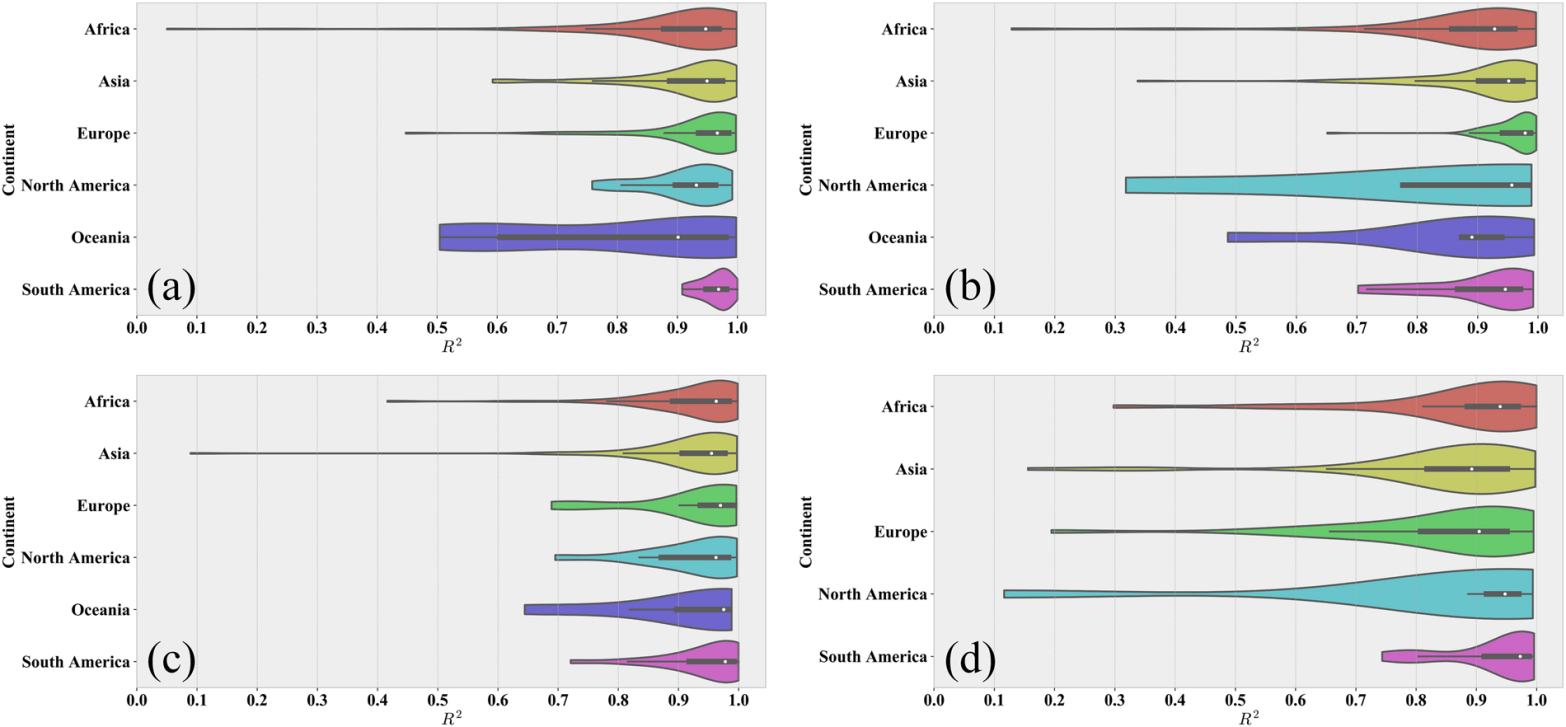
Violin plots of model performance (R²) across continents for (**a**) Maize; (**b**) Wheat; (**c**) Rice; (**d**) Soybean.

Comparing the continents, we found that crop model performance in Europe and South America is generally the best. Particularly in South America, the model performance for all four crops is relatively stable, with a small variation range. In contrast, the performance in Africa and Oceania is relatively poor, which may reflect the influence of different agricultural models and agricultural stability on the difficulty of modeling. For example, the large-farm model in Europe and South America may be more conducive to remote sensing data capturing crop growth information, thereby improving model performance. Meanwhile, the scattered and limited maize cultivation area in Oceania results in low R² due to the low spatial resolution and mixture of different crop types. In Africa, smallholder agriculture is dominant, and agricultural production is greatly affected by natural conditions. The unstable agricultural conditions in Africa make modeling difficult, so the R² variation range of the models for all four crops is relatively large.

Model performance for the same crop also varies across continents. For instance, wheat models perform best in Europe (average R² up to 0.96) and relatively lower in North America (average R² of 0.81). One reason is that the spatial scale of modeling units in Europe is much smaller than in North America, so the models can more easily capture crop growth conditions and yield changes within the modeling units, thus improving estimation accuracy. For rice, the R² variation range is the largest in Asia, which may be due to the complex cropping systems, diverse environmental conditions, and relatively fragmented planting areas in Asian rice-growing regions all increase the difficulty of modeling.

Overall, the performance of models for different crops in all continents is relatively good, with most models having an R² above 0.8, indicating that the models trained in this study have high accuracy and reliability in different regions. These analyses also reflect some patterns, i.e., modeling difficulty is significantly associated with regional agricultural characteristics (such as agricultural models, cropping systems, natural conditions, etc.). The more stable the agricultural conditions, the more standardized the planting patterns, and the lower the fragmentation of planting areas, the easier it is for remote sensing data to accurately capture crop growth status, and the better the performance of crop production estimation models. In regions with unstable agricultural conditions, diverse planting patterns, and high degrees of planting area fragmentation, yield variability is large, and the difficulty of extracting remote sensing information is high, resulting in relatively poor model performance.

In the future, for regions with relatively lower performance, we can consider introducing more influencing factors that reflect regional agricultural characteristics and optimizing model algorithms to improve the regional adaptability of crop production estimation models. At the same time, obtaining more detailed regional cropping system information will also help improve the performance of regionalized models.

### Comparing with existing datasets

To comprehensively validate the GGCP10 dataset, we conducted systematic comparisons against multiple reference sources: global gridded datasets, regional gridded products, and extensive subnational statistics. This multi-tiered validation approach encompasses different spatial scales and geographical contexts, providing a robust assessment of GGCP10’s reliability and accuracy.

Our validation framework consists of three components. First, we compared GGCP10 with two established gridded datasets: the global SPAM 2010 dataset and the regional AsiaRiceYield4KM dataset, enabling pixel-by-pixel evaluation of spatial patterns. Second, we assembled an extensive collection of subnational statistics from eight diverse sources worldwide, including data from Africa, Europe, North America, South America, Asia, and Australia, covering various administrative levels from county to province. This comprehensive subnational validation spans 2,823 administrative units across 43 countries. Third, we performed detailed spatial analysis using county-level USDA survey data to examine local-scale accuracy in a major agricultural region.

While acknowledging that reference datasets may contain their own uncertainties and limitations in temporal coverage or spatial resolution, this comprehensive validation approach allows us to evaluate GGCP10’s performance across different scales, regions, and agricultural systems. The consistency analysis includes both quantitative metrics (correlation coefficient (Corr), coefficient of determination (R²), and root mean square error (RMSE)) and detailed spatial pattern comparisons, providing users with clear understanding of the dataset’s strengths and limitations across different geographical contexts and production scales.

**Fig. 12:**
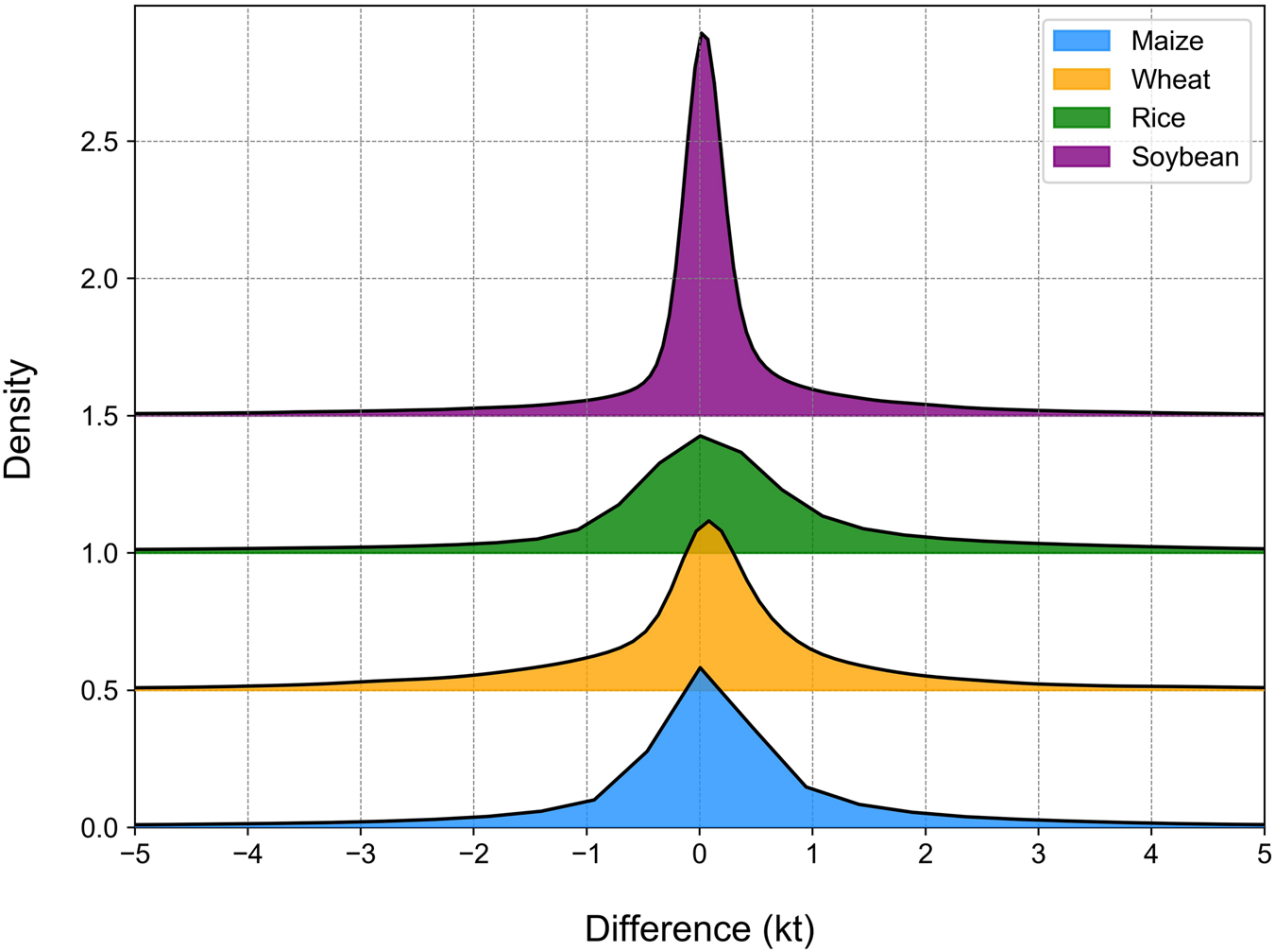
The density distributions of the differences between GGCP10 and SPAM 2010.
  Figure 12 shows the density distributions of the differences between GGCP10 and SPAM 2010 for each crop. The curves were centered around zero, indicating overall consistency. Soybean shows the highest agreement, with 78.2% of pixels having differences between -1 and 1 kiloton, and only 2.5% of pixels with differences less than -5 or greater than 5 kilotons. Maize and wheat also demonstrate good alignment, with around 65% of pixels having differences within ± 1 kiloton. Rice exhibits relatively lower consistency, with 60.3% of pixels within ± 1 kiloton difference and 13.2% of pixels showing differences beyond ± 5 kilotons. The mean differences for all crops are close to zero (-0.01 to -0.18 kilotons), further confirming the general agreement between GGCP10 and SPAM 2010. However, there was a slight tendency for GGCP10 to overestimate compared to SPAM 2010, as indicated by the lower percentages of pixels with negative differences (ranging from 33.9% for soybean to 44.0% for rice).
  To provide a more informative comparison, we conducted a grid-to-grid analysis between GGCP10 and SPAM 2010 for maize, wheat, rice, and soybean. Figure 13 shows the scatter density plots of this comparison, with both axes logarithmically scaled to better visualize the wide range of production values.

1. **Comparison with SPAM 2010**. To assess the reliability of GGCP10, we compared it with the widely used global agricultural production dataset SPAM 2010^9^ for four major crops: maize, wheat, rice, and soybean. Because SPAM 2010 and GGCP10 use different units (tons and kilotons, respectively), we converted the SPAM 2010 data to kilotons for consistent comparison. We generated overlapping density curves and calculated the key statistics to evaluate the agreement between the two datasets, as shown in Fig. 12.

**Fig. 13:**
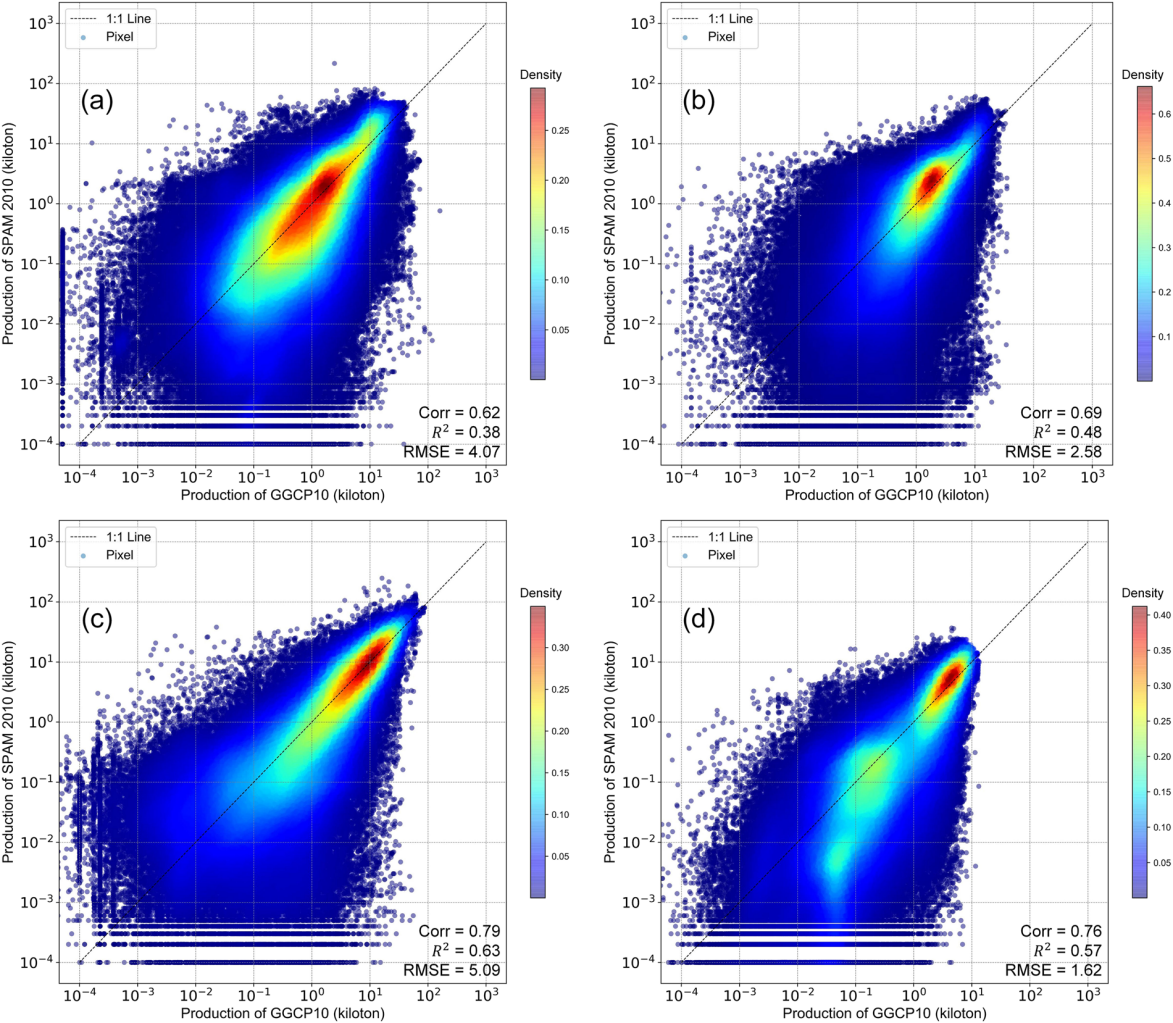
Log-log scatter density plots comparing GGCP10 and SPAM 2010 production estimates (kilotons per 10 km grid) for (**a**) maize, (**b**) wheat, (**c**) rice, and (**d**) soybean. Both x and y axes are logarithmically (base 10) transformed to better visualize the wide range of production values. The color scale represents the density of points, with warmer colors indicating higher densities. The dashed line represents the 1:1 relationship.
  The grid-to-grid comparison between GGCP10 and SPAM 2010 reveals strong positive correlations for all four crops, with Corr ranging from 0.62 (maize) to 0.79 (rice). The scatter density plots demonstrate that most grid cells have similar production estimates in both datasets, particularly for medium to high production values. Rice shows the strongest agreement with the highest correlation and R² values, while soybean exhibits the lowest RMSE. Wheat and maize display good overall consistency, with wheat showing tighter clustering around the 1:1 line compared to maize.
  Despite the general agreement, there are noticeable differences between the datasets, particularly for lower production values where greater dispersion is observed. The RMSE values, ranging from 1.62 (soybean) to 5.09 (rice) kilotons, indicate varying levels of local differences between GGCP10 and SPAM 2010. These findings suggest that while GGCP10 captures similar spatial patterns of crop production as SPAM 2010, there is room for continued refinement and validation, especially in areas with lower production values.
  To illustrate the consistency and differences between GGCP10 and SPAM 2010, we selected key regions, including Africa (maize), Western Europe (wheat), Southeast Asia (rice), Brazil, and Argentina in South America (soybean). The results are presented in Fig. 14.

**Fig. 14:**
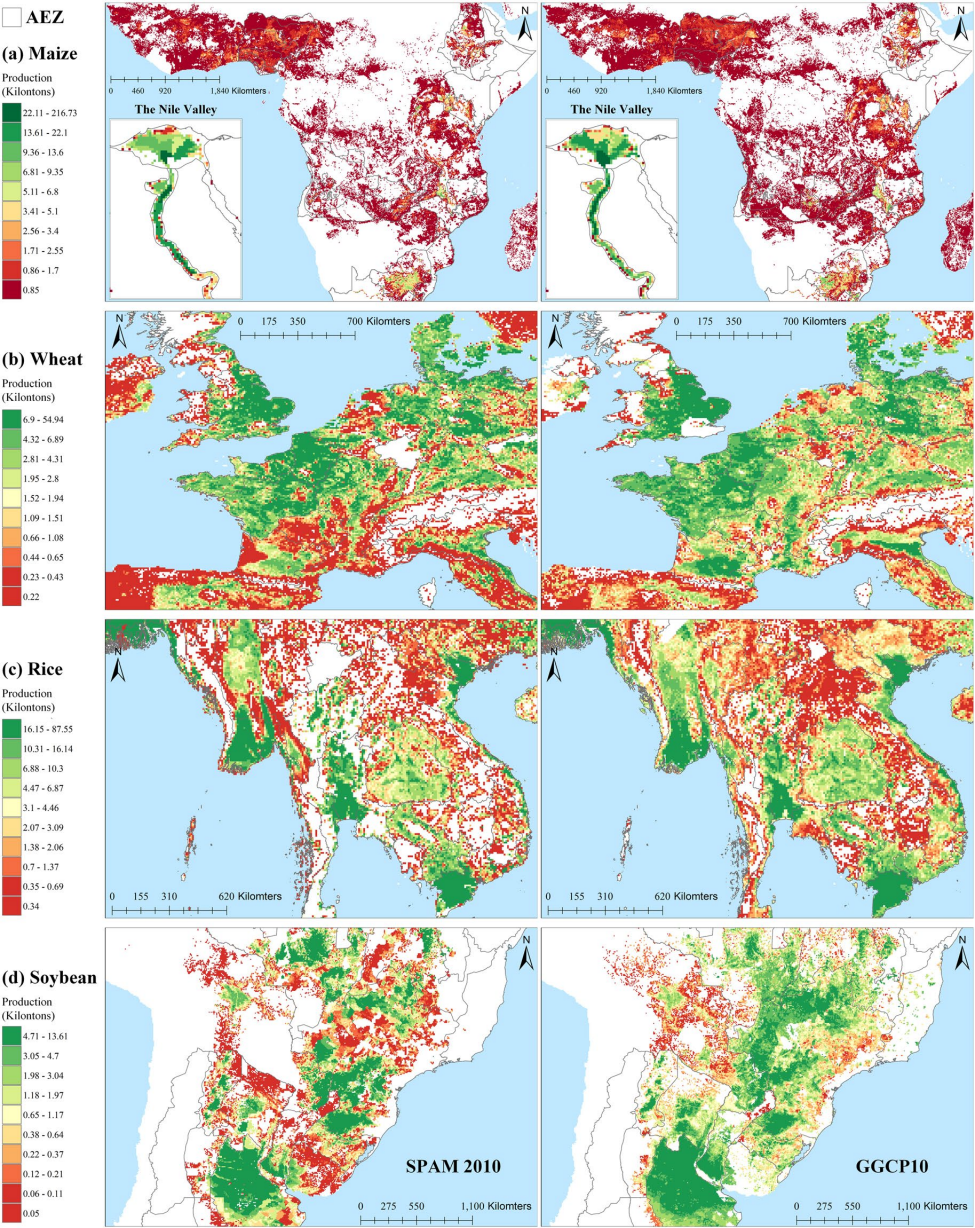
Spatial comparison of crop production between SPAM 2010 and GGCP10 datasets for selected regions. (**a**) Maize production in Africa; (**b**) Wheat production in Western Europe; (**c**) Rice production in Southeast Asia; (**d**) Soybean production in Brazil and Argentina, South America.
  Owing to differences in the definition of arable land between the two datasets, inconsistencies in the covered pixels are inevitable. From a detailed perspective, the two datasets have very high consistency in high-production regions, whereas the inconsistent regions are mainly located in low-value areas. In addition, compared with SPAM2010, GGCP10 exhibited smoother spatial transitions.
  In summary, the comparison revealed a high level of consistency between GGCP10 and SPAM 2010, particularly for soybean, maize, and wheat. The agreement was slightly lower for rice, but still within an acceptable range. These findings support the reliability of the GGCP10 as a global gridded crop production dataset.
2. **Comparison with AsiaRiceYield4km**. The AsiaRiceYield4km^48^ dataset provides a high-resolution (4 km) seasonal grid of rice yields in Asia spanning 1995 to 2015 and covers single-, double-, and triple-season rice. To harmonize the evaluation metrics and ensure consistency with our GGCP10 dataset, several adjustments were made to the AsiaRiceYield4km dataset.
  Owing to the unavailability of seasonal harvested area data in AsiaRiceYield4km, the comparison was constrained to single-season rice areas, which constitute 56.5% of the total AsiaRiceYield4km area. To align with the spatial resolution of GGCP10, the AsiaRiceYield4km dataset was resampled to a 10 km grid. During the development of the GGCP10 dataset, we generated the corresponding harvested area data, allowing us to calculate the total production values for AsiaRiceYield4km based on these areas. The recalculated total production data served as the basis for consistency evaluation with the GGCP10 dataset.
  For the overlapping years from 2010 to 2015, scatter density plots (Fig. 15) were used to assess the consistency of gridded production data between the GGCP10 and AsiaRiceYield4km.

**Fig. 15:**
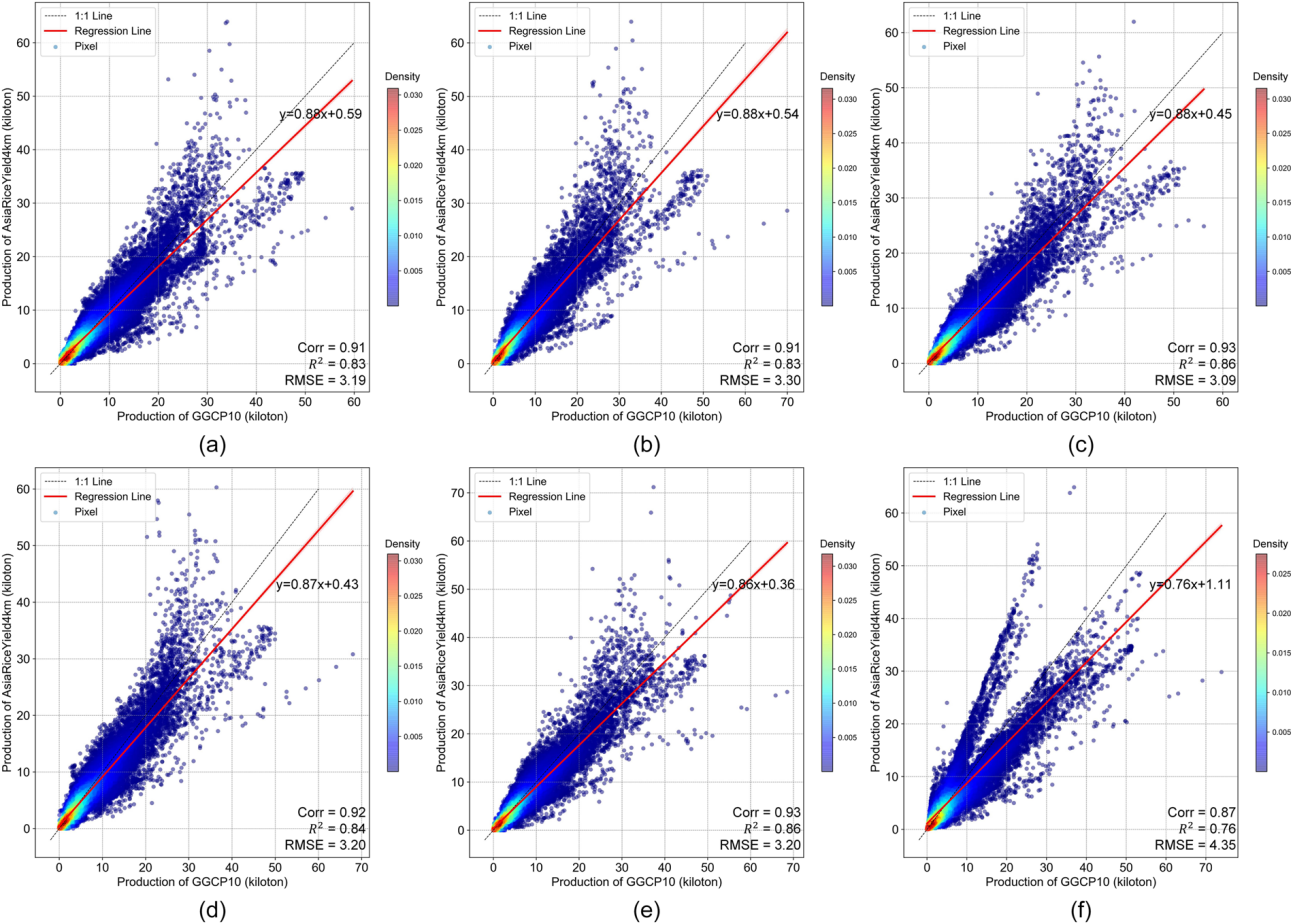
Scatter density plots comparing GGCP10 and AsiaRiceYield4km rice production estimates (kilotons per 10 km grid) for years. (**a**) 2010; (**b**) 2011; (**c**) 2012; (**d**) 2013; (**e**) 2014; (**f**) 2015.
  The data revealed a strong positive correlation between the GGCP10 and AsiaRiceYield4km for the years 2010–2014. The data points closely align along the 1:1 line, reinforcing the idea that GGCP10 accurately captures the data distribution patterns present in AsiaRiceYield4 km. Acceptable error rates are indicated by RMSEs ranging from 3.09 to 3.30 kilotons per 10 km grid. The R² values range from 0.83 to 0.86, and correlation coefficients are between 0.91 and 0.93. Notably, 2015 exhibited a marginally lower slope and R², but remained within acceptable limits. It should be noted that for all years examined, the slope of the fitted line was less than 1, suggesting that GGCP10 tends to overestimate production when compared to AsiaRiceYield4km.
  In summary, the GGCP10 exhibits a strong degree of consistency with AsiaRiceYield4km in terms of single-season rice production grids, although localized discrepancies warrant further investigation.
3. **Comparison with global subnational statistical data**. To comprehensively validate the GGCP10 dataset on a global scale, we collected eight sets of subnational crop production statistics covering diverse regions worldwide (Table 3). These datasets are:

**Table 3:** Summary of subnational statistical datasets used for global validation.

Data source	Unit type	Crop type coverage	Unit coverage	Time coverage
HarvestStat Africa	Mixed (Province / City)	Maize	331 units of 27 countries	2010–2020
		Wheat	79 units of 12 countries	2010–2020
		Rice	249 units of 26 countries	2010–2020
		Soybean	90 units of 11 countries	2010–2020
Harmonized European Union subnational crop statistics	NUTS	Maize	387 units of 11 countries	2010–2020
		Wheat	769 units of 16 countries	2010–2020
USDA “SURVEY” data of USA	County	Maize	1998 units of USA	2010–2020
		Wheat	1644 units of USA	2010–2020
		Rice	87 units of USA	2010–2020
		Soybean	1706 units of USA	2010–2020
Agricultural statistics from Directorate of Economics and Statistics of the Department of Agriculture of India	State	Maize	28 units of India	2010–2020
		Wheat	23 units of India	2010–2020
		Rice	30 units of India	2010–2020
		Soybean	20 units India	2010–2020
Agricultural statistics from National Bureau of Statistics of China	Province	Maize	31 units of China	2010–2020
		Wheat	30 units of China	2010–2020
		Rice	30 units of China	2010–2020
		Soybean	30 units of China	2010–2020
Agricultural statistics from Ministry of Agriculture, Livestock and Fisheries of Argentina	Province	Maize	15 units of Argentina	2010–2020
		Wheat	14 units of Argentina	2010–2020
		Rice	5 units of Argentina	2010–2020
		Soybean	15 units of Argentina	2010–2020
Agricultural statistics from Central Statistics Agency of Indonesia	Province	Maize	33 units of Indonesia	2010–2015
		Rice	33 units of Indonesia	2010–2015 2018–2020
Agricultural statistics from Australian Bureau of Statistics	State	Wheat	4 units of Australia	2017–2020

1. HarvestStat Africa^49^
2. Harmonized European Union subnational crop statistics^50,51^
3. USDA “SURVEY” data of USA (https://quickstats.nass.usda.gov)
4. Agricultural statistics from Directorate of Economics and Statistics of the Department of Agriculture of India (https://data.desagri.gov.in/website/crops-apy-report-web)
5. Agricultural statistics from National Bureau of Statistics of China (https://data.stats.gov.cn/english/easyquery.htm?cn=E0103)
6. Agricultural statistics from Ministry of Agriculture, Livestock and Fisheries of Argentina (https://datosestimaciones.magyp.gob.ar/reportes.php?reporte=Estimaciones)
7. Agricultural statistics from Central Statistics Agency of Indonesia (https://www.bps.go.id/en/statistics-table?subject=557)
8. Agricultural statistics from Australian Bureau of Statistics (https://www.abs.gov.au/statistics/industry/agriculture/agricultural-commodities-australia)

Taking maize as an example, these datasets encompass 2,823 subnational units across 43 countries at various administrative levels. This extensive coverage allows for a comprehensive assessment of GGCP10’s performance across diverse agricultural systems and geographies.

**Fig. 16:**
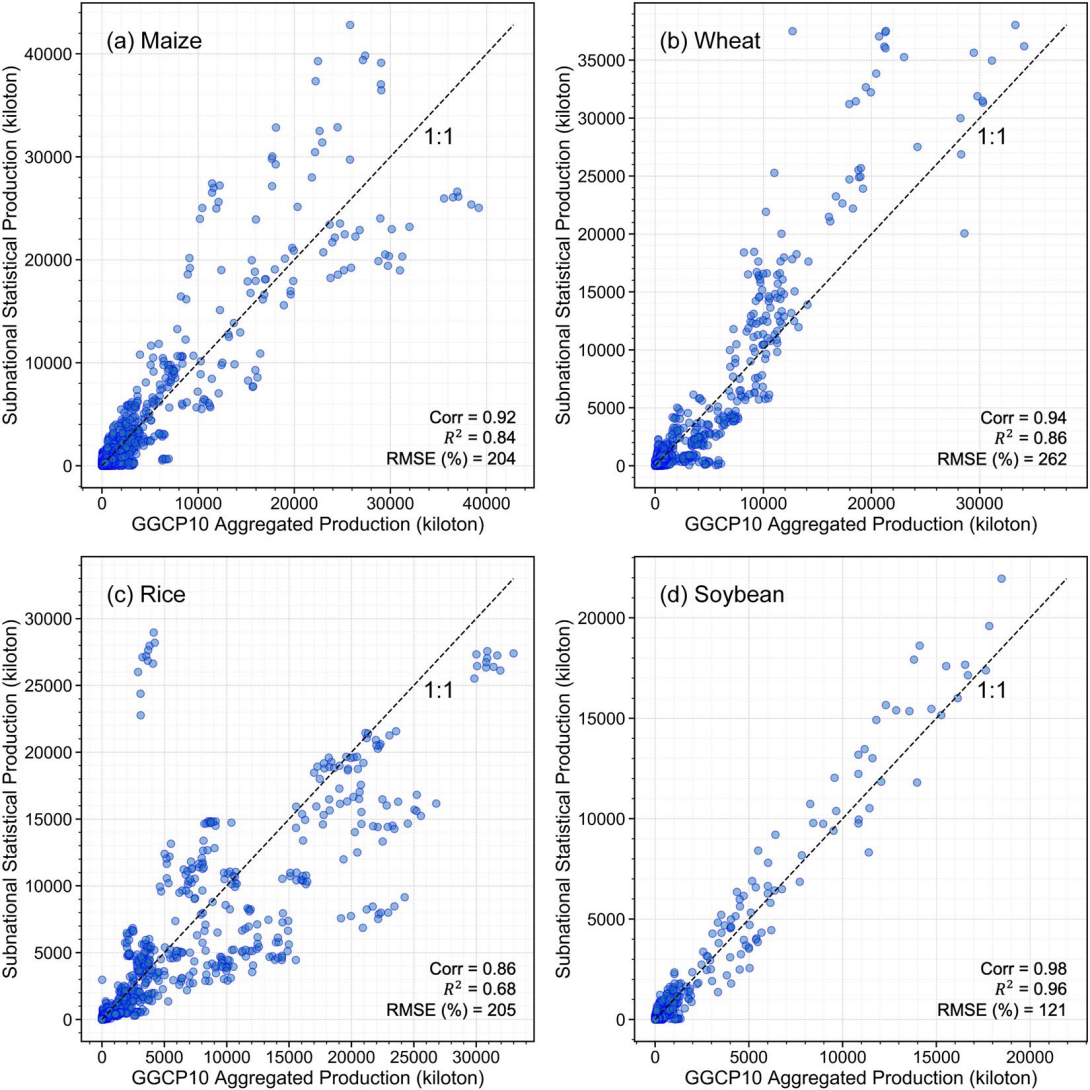
Comparison of GGCP10 aggregated crop production with subnational statistical data. Scatter plots are shown for (**a**) maize, (**b**) wheat, (**c**) rice, and (**d**) soybean. The dashed line represents the 1:1 relationship. RMSE values are expressed as percentages of mean production.

To conduct the comparison, we aggregated GGCP10 data based on the boundaries of each subnational unit. We then created scatter plots (Fig. 16) comparing GGCP10 aggregated production estimates with subnational statistical data for maize, wheat, rice, and soybean. These plots offer a quantitative assessment of the dataset’s performance across different crops and production scales.

The scatter plots reveal varying levels of agreement between GGCP10 estimates and subnational statistics across the four crops. Maize shows a strong positive correlation (Corr = 0.92, R² = 0.84), with data points clustering closely around the 1:1 line, indicating good overall agreement. Wheat demonstrates even higher correlation (Corr = 0.94, R² = 0.86), with tightly clustered data points, though there’s a slight underestimation trend for high-production areas. Rice exhibits the lowest correlation among the four crops (Corr = 0.86, R² = 0.68), with a more dispersed pattern, especially for production levels between 5,000 and 25,000 kilotons where GGCP10 tends to overestimate. Soybean shows the strongest performance with the highest correlation (Corr = 0.98, R² = 0.96) and the lowest RMSE (121%), indicating excellent agreement between GGCP10 and subnational statistics across all production levels. RMSE values for maize (204%), wheat (262%), and rice (205%) suggest significant variability, particularly for smaller production units.

Despite some variations, the high correlations and R² values across all crops indicate that GGCP10 provides reliable estimates of crop production at the subnational level, particularly for larger production units.

**Fig. 17:**
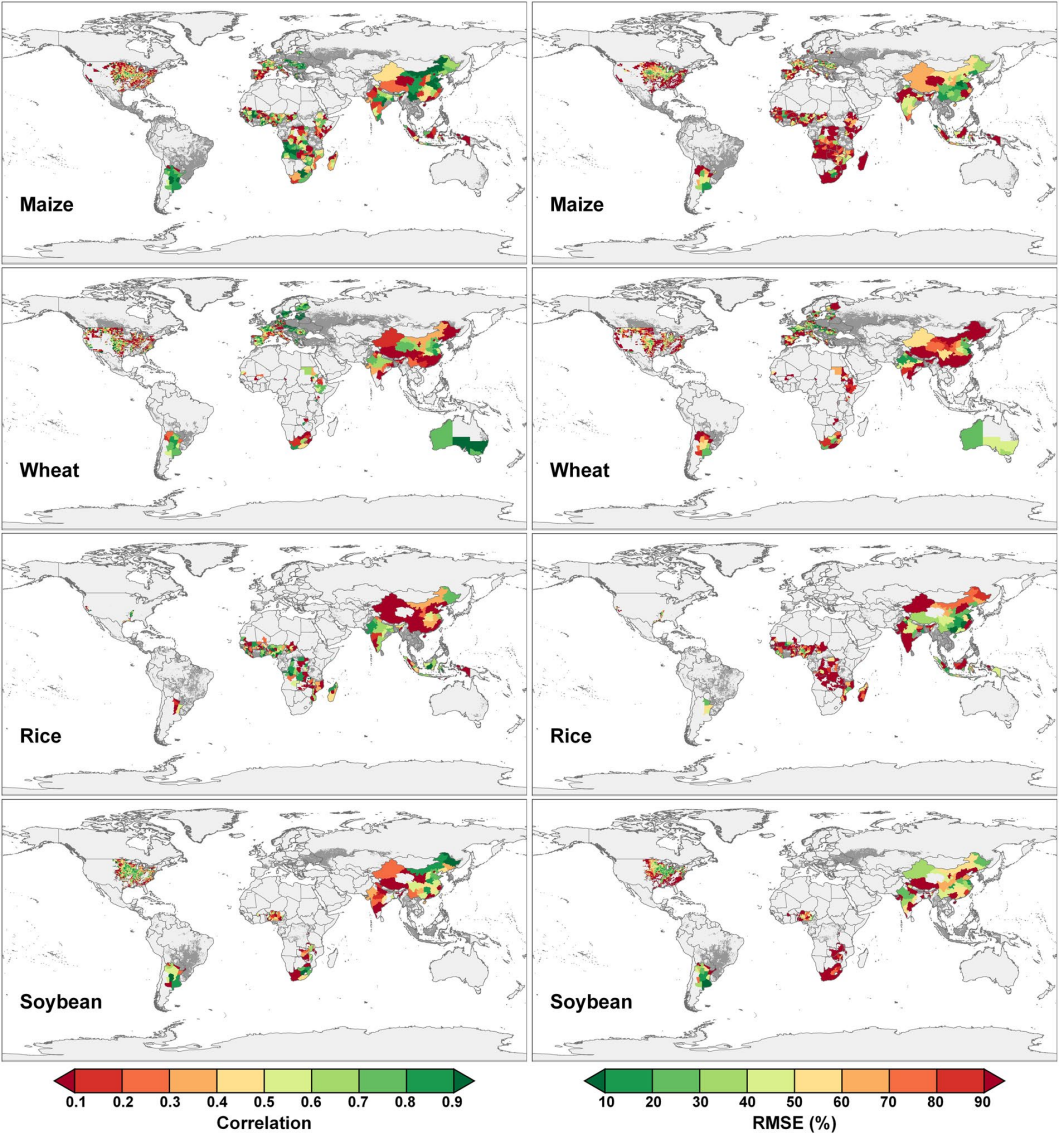
Global comparison of GGCP10 crop production with subnational statistics. The left column displays correlation coefficients, while the right column shows RMSE percentages for maize, wheat, rice, and soybean. Gray areas represent background regions, while dark gray areas indicate regions with valid GGCP10 data coverage.

We then calculated two metrics for each unit: the correlation coefficient and RMSE (relative to the unit’s mean production in 2010–2020). A global consistency comparison map was created for the four major crops (maize, wheat, rice, and soybean), providing a comprehensive visual assessment of GGCP10’s accuracy across different geographical areas and agricultural systems (Fig. 17).

Figure 17 presents a comprehensive global assessment of GGCP10’s performance for four major crops (maize, wheat, rice, and soybean) at the subnational level. For maize, high correlations (>0.7) and low RMSE percentages (<30%) are observed in major producing regions such as the U.S. Corn Belt, China’s Northeast Plain and North China Plain, Argentina’s Pampas, and India’s Gangetic Plain, indicating strong agreement between GGCP10 and official statistics in these key areas. Wheat shows robust performance in major wheat-growing regions, with strong correlations and low RMSE percentages evident in the U.S. Midwest, most of Western Europe, Australia, Argentina’s Pampas, Northern China, and Northern India. Rice estimates demonstrate high accuracy in key producing areas of Asia, including Indonesia, India’s Ganges Plain, China’s Heilongjiang Province, and West Africa’s Gulf of Guinea, as reflected by high correlations and lower RMSE percentages. Soybean production patterns are effectively captured in major producing regions, with strong correlations and low RMSE percentages in Argentina’s Pampas, the U.S. Mississippi River Basin and Central Plains, China’s Northeast Plain, and South Africa’s semi-arid grasslands.

Overall, GGCP10 demonstrates high consistency with subnational statistical data in global major production areas for all four crops. The dataset shows robust performance in capturing production patterns in areas with well-established agricultural systems and data infrastructure, particularly in North America, Europe, and large parts of Asia. However, the correlation and RMSE values gradually worsen as regional production values decrease. Higher RMSE percentages and lower correlations are observed in parts of Africa, non-major producing provinces in China, Spain within the EU, and non-major producing counties in the United States.

**Fig. 18:**
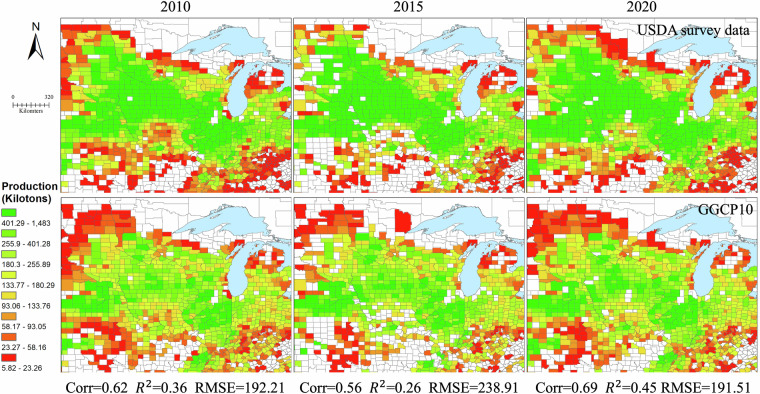
Spatial comparison of county-level maize production (kilotons) between GGCP10 and USDA survey data for 2010, 2015, and 2020.

**Fig. 19:**
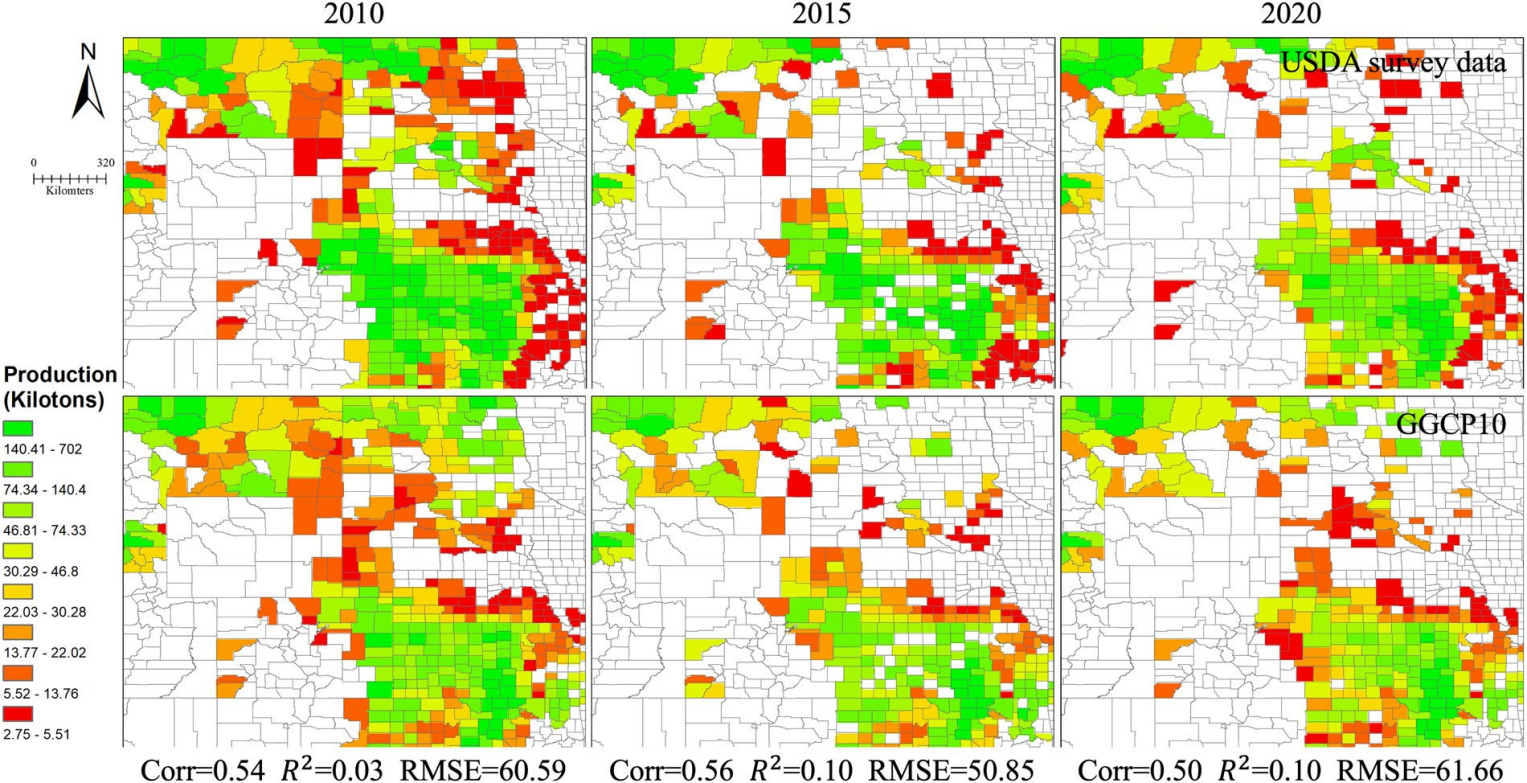
Spatial comparison of county-level wheat production (kilotons) between GGCP10 and USDA survey data for 2010, 2015, and 2020.

**Fig. 20:**
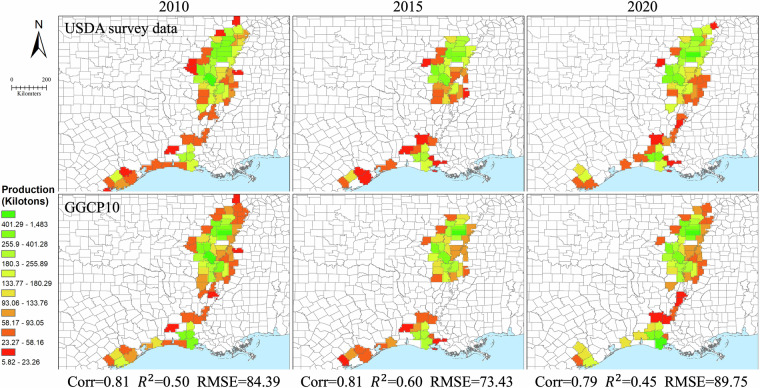
Spatial comparison of county-level rice production (kilotons) between GGCP10 and USDA survey data for 2010, 2015, and 2020.

**Fig. 21:**
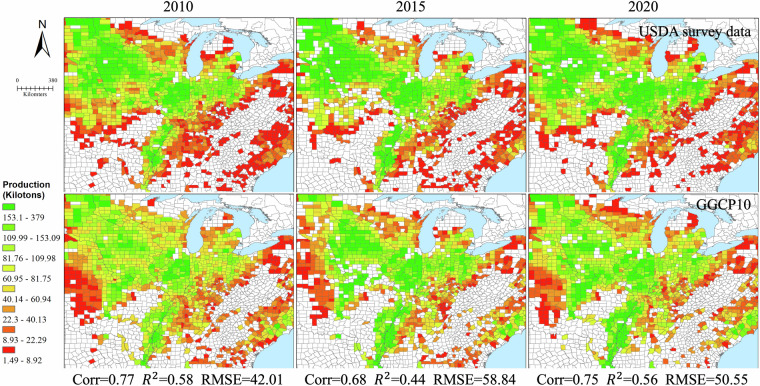
Spatial comparison of county-level soybean production (kilotons) between GGCP10 and USDA survey data for 2010, 2015, and 2020.

Furthermore, to more clearly illustrate the spatial distribution details, considering that the USDA data is the most fine-grained (county level) among the aforementioned subnational datasets, we selected this dataset for a detailed spatial comparison with GGCP10 at the county level. To assess the consistency between GGCP10 and the USDA data, we selected data from three years (2010, 2015, and 2020). We aggregated the GGCP10 dataset based on the boundaries of counties in the United States to obtain production data at the county level. We then performed spatial visualization of the two datasets to intuitively present their consistency and differences (Figs. 18, 19, 20 and 21), and calculated corresponding accuracy metrics (Corr, R², RMSE). It should be noted that, to focus on the main crop-producing areas and improve the readability of the maps, we only display the primary regions in the figures.

For maize, the spatialization results showed that, compared to the USDA data, the underestimation of the GGCP10 dataset was mainly located in North Dakota, and the overestimation was mainly located in Kentucky, whereas for other regions, the spatial distribution consistency of the two datasets was relatively high. Overall, GGCP10 tends to overestimate production in low-production areas and underestimate production in high-production areas compared to the USDA survey data.

For wheat, although the R² values were relatively low, the lower RMSE and higher correlation coefficients indicated significant correlations between GGCP10 and the USDA data. The spatialization results show that, compared to the USDA data, the GGCP10 dataset shows a relatively obvious underestimation in South Dakota and Nebraska, whereas overestimation is more evident in North Dakota. Apart from those regions, the other regions maintained good consistency.

For rice, the spatialization results showed that there were relatively few regions cultivating rice in the United States. In terms of accuracy metrics, correlation and R² were significantly higher than for maize and wheat; and the spatial consistency between the USDA data and the GGCP10 dataset was very high, with overestimation occurring only in some counties.

For soybean, GGCP10 showed high consistency with the USDA dataset. The spatialization results showed that the underestimation of high-production areas by the GGCP10 dataset was mainly located in Indiana, South Dakota, and Nebraska, whereas the consistency in other high-production states, such as Illinois, was relatively high. The overestimated areas were mainly located in North Carolina, Wisconsin, and Minnesota in 2010.

All four crops had relatively high correlation coefficients, indicating good agreement between our dataset and the USDA data. Although R² was relatively low for wheat, this may be due to potential sampling bias, as the USDA dataset was derived from sample surveys. Overall, our dataset showed high consistency with the USDA data, demonstrating its higher reliability and reference value.

## Usage Notes

The GGCP10 dataset, while comprehensive and valuable for global crop production analysis, has certain inherent limitations and potential sources of uncertainty that users should consider when applying it to their research or decision-making processes. The following paragraphs elucidate these key considerations to ensure appropriate interpretation and application of the data.

1. **Limitation in using FAO national statistical data**. FAO statistical data, while an important source for the GGCP10 dataset, have limitations due to their coarse spatial resolution and potential variability in data quality across countries. These factors may lead to inconsistencies between GGCP10 and local statistics in some regions. While subnational crop statistics could potentially improve modeling accuracy, the lack of globally unified sub-regional data and significant variations in data characteristics across countries make their incorporation challenging without compromising global comparability.
  Despite these limitations, FAO national agricultural statistics offer clear advantages for global-scale production estimations. Their authoritative nature, long time series, and systematic verification process provide a consistent benchmark across countries. By using FAO data for calibration, GGCP10 maximizes the use of existing data resources while ensuring consistency in global benchmarks. This approach enables the dataset to better serve applications such as monitoring Sustainable Development Goals (SDGs) and assessing global food security, albeit at the cost of some subnational detail.
2. **Uncertainties in crop spatial distribution**. The crop spatial distribution information in the GGCP10 was based on reference data from 2015. However, the spatial allocation of crop planting areas may differ across years owing to factors such as agricultural policies, market demands, and climate change. This may introduce uncertainties in the crop distribution for certain grids, consequently affecting our estimates of the harvested area and crop production^52^. Although the comparison with other datasets showed a high level of consistency, there were instances of overestimation or underestimation in certain regions or for certain crops, which may have been caused by this uncertainty.
  The irrigation datasets were considered in model development, however, cropland could be cultivated at different cropping intensity across the region and across years. Considering the availability of the cropping intensity at global scales either at low resolution or high resolution^53–55^, we will further enhance the production estimation model by integration of the cropping intensity dataset.
3. **Implications for Data Users**. When faced with these limitation and uncertainties in the GGCP10 dataset, users should exercise caution when processing and interpreting the results of related research.

The GGCP10 dataset was calibrated for consistency with FAO national-level statistics; however, the data sources for different countries primarily came from their respective agencies. This implies that the reliability of statistical data may vary by country, and that such regional differences may influence the conclusions drawn from cross-national or large-scale comparative analyses.

Moreover, if users have access to more refined statistical data for their regions of interest, we recommend that they perform a secondary calibration of their initial estimation results using this more reliable regional data. Specifically, users can calculate the ratio coefficient between our initial estimates and the regional statistical totals, and then use this coefficient to proportionally scale the gridded production data to match the regional statistical totals.

Additionally, due to differences in resolution, definitions of cropland extent, and other factors, the yield values calculated from the GGCP10 dataset may differ from those obtained through ground surveys. These discrepancies could potentially lead to the overestimation or underestimation of yield-influencing factors when using the GGCP10 dataset in research or decision-making processes. Users should be aware of these potential limitations and interpret the results with caution.

## Data Availability

The custom code used for generating and processing the GGCP10 dataset is publicly available on GitHub: https://github.com/QinXingli/GGCP10_Method.git. This repository contains all custom code used in our research. The code is written in Python 3.7. For long-term preservation and to enable citation, we have archived the specific version of the code used in this study on Zenodo^56^ with the following 10.5281/zenodo.13626322. This archived version corresponds to GitHub release v1.0.0. The code is freely accessible to anyone for use, modification, and distribution, provided appropriate credit is given. There are no restrictions on access. The repository includes a detailed README file with instructions for installation and usage.
